# Crohn’s Disease Exclusion Diet for Remission Induction in Adults: A Systematic Review and Stratified Synthesis

**DOI:** 10.3390/nu18213656

**Published:** 2026-11-03

**Authors:** Jia Zhang, Xi Yuan, Qingying Luo

**Affiliations:** College of Food Science, Sichuan Agricultural University, Ya’an 625014, China; zhangjahello@gmail.com (J.Z.); tiffaniguayxx1256@gmail.com (X.Y.)

**Keywords:** Crohn’s disease, Crohn’s disease exclusion diet, dietary therapy, nutritional intervention, partial enteral nutrition, systematic review

## Abstract

**Background/Objectives**: The Crohn’s disease exclusion diet (CDED) offers a structured nutritional strategy for adults with Crohn’s disease. We evaluated remission outcomes by comparator, assessment time, and analysis population to clarify its clinical interpretation. **Methods**: Six databases were searched from January 2014 to 2 September 2026. Week-6 clinical remission was primary; later assessments were secondary. Randomized comparisons were analyzed separately by comparator and time point. Uncontrolled proportions were reported individually using exact binomial confidence intervals. Risk of bias and certainty were assessed using design-specific tools. The protocol was registered in PROSPERO (CRD420261381711), with broader activity-range inclusion and exploratory analyses reported as deviations. **Results**: Seven studies comprised two randomized trials and five uncontrolled studies. At week 6, remission occurred in 13/19 participants receiving CDED plus partial enteral nutrition versus 12/21 receiving CDED alone (risk ratio 1.20, 95% confidence interval 0.74–1.94). A separate trial favored CDED over a Mediterranean diet at week 12 (17/24 versus 8/21; 1.86, 1.02–3.40). Comparative certainty was very low or low. Descriptive, exploratory uncontrolled week-6 remission proportions in clinically active populations were 30/48 (62.5%) and 23/30 (76.7%). Objective inflammatory outcomes were inconsistently assessed and showed no consistent comparative advantage accompanying symptomatic remission. **Conclusions**: Adult evidence identifies a symptomatic remission signal that differs by comparator and assessment time. Comparative biochemical or endoscopic superiority remains unestablished. This synthesis clarifies the population and treatment question represented by each estimate. Trials should assess inflammatory control, nutritional adequacy, adherence, and longer-term outcomes during treatment and after cessation.

## 1. Introduction

Dietary treatment offers an adjunctive or alternative induction strategy for Crohn’s disease (CD), linking disease management with nutritional care. Current nutrition and inflammatory bowel disease (IBD) guidance supports individualized decisions based on disease activity, patient preferences, and specialist nutritional support [1,2,3]. Exclusive enteral nutrition (EEN) has a stronger evidence base in children, whereas adherence and acceptability can limit its use in adults. The Crohn’s disease exclusion diet (CDED) combines selected ordinary foods with restrictions on specific dietary components. Adult evaluation is therefore needed to account for treatment histories and nutritional needs that may differ from those of children.

Experimental studies provide a rationale for restricting selected processed-food components. Emulsifiers altered microbial communities and promoted inflammation in animal models; maltodextrin enhanced bacterial adhesion or impaired the mucus barrier [4,5,6]. κ-Carrageenan also aggravated experimental colitis [7]. These findings motivate clinical evaluation of the dietary approach. A pediatric trial found better tolerance of CDED plus partial enteral nutrition (PEN) than EEN, with concurrent clinical and biological improvements [8]. Adult studies are needed to establish the clinical relevance of this approach in a different treatment setting.

Previous reviews examined CDED across age groups or evaluated several solid-food interventions [9,10,11,12]. Batra et al. also reported an adult subgroup within a broader CDED meta-analysis [12]. These syntheses establish the wider evidence base, while adult treatment decisions require closer attention to comparators, assessment times, and analysis populations. The first CDED report, for example, included 13 adults within a mixed-age cohort and used a stricter remission threshold in a treatment-experienced subgroup [13]. Disease duration, biologic exposure, background medication, and dietary delivery further shape the interpretation of adult outcomes.

We evaluated CDED, alone or with PEN, for remission induction in adults with CD, incorporating the updated evidence through 2 September 2026. We distinguished the incremental contribution of PEN from comparison with another diet, separated week-6 induction from later assessments, and examined outcome-specific analysis populations. We also assessed whether symptom-defined remission was accompanied by biochemical or endoscopic improvement. Together, these distinctions identify the adult treatment question answered by each study and the evidence relevant to nutritional practice.

## 2. Materials and Methods

### 2.1. Protocol, Search, and Reporting

This systematic review followed the Preferred Reporting Items for Systematic Reviews and Meta-Analyses (PRISMA) 2020 statement [14]. The protocol was registered in PROSPERO (CRD420261381711) on 28 April 2026. The registration was subsequently updated to record the search extension to 2 September 2026 and the inclusion of Dileo et al. PubMed, Embase via Ovid, the Cochrane Library, Web of Science All Databases, Scopus, and China National Knowledge Infrastructure were searched. Searches covered 1 January 2014 to 2 September 2026; the starting year corresponds to the first clinical report of the named, structured CDED intervention [13]. Database-specific strategies, platforms, restrictions, and record counts are provided in Supplementary Table S1.

Comparative results were stratified by comparator because each comparison was supported by one trial. The exploratory single-arm syntheses were not prospectively registered. During revision, the initial pooling approach was replaced by individual-study reporting because activity definitions, recruitment, and analysis populations differed. The post hoc missing-outcome scenario for Szczubełek was retained at the study level. Departures from the initial activity-range target are described below. This review used published aggregate data without new participant recruitment or identifiable individual-level information.

### 2.2. Eligibility and Applicability

Eligible designs included randomized controlled trials (RCTs), prospective or retrospective cohorts, and single-arm intervention studies reporting adult CD outcomes with CDED, alone or with PEN. Adult data from mixed-age reports had to be separately extractable for quantitative synthesis. Comparative evidence was organized into two specified strata: CDED + PEN versus CDED alone, and CDED versus the Mediterranean diet (MD). Uncontrolled studies of CDED with or without PEN required no parallel comparator. Prespecified outcome categories included clinical remission or response, inflammatory biomarkers, endoscopic or imaging findings, adherence, discontinuation, and safety.

The initial target was adults aged ≥18 years with mild-to-moderate active luminal CD, defined by Harvey–Bradshaw Index (HBI) 5–8 or Crohn’s Disease Activity Index (CDAI) 150–450. Montreal B1 or non-obstructive B2 behavior was the intended clinical context. Yanai and Pasta enrolled broader ranges, HBI 5–14 and 5–16, respectively, without separately extractable results for HBI 5–8. Their whole-study estimates were retained as an explicit protocol deviation to preserve the available randomized adult comparisons. Estimates were interpreted for the populations enrolled. HBI and CDAI ranges were not assumed to represent identical severity. Disease duration, prior therapy, and concomitant medication were recorded as applicability factors in Table 1 and Supplementary Table S10.

Studies without separately extractable adult data were excluded. Other excluded populations included perioperative disease, acute obstruction, penetrating disease requiring surgery, severe disease or malnutrition, and pregnancy when target-population data could not be separated. Animal studies, reviews, conference abstracts, and duplicate reports were excluded from the primary evidence set. Mixed induction/maintenance populations, stricter remission thresholds, and special treatment contexts were recorded separately. Mixed-age findings and broader cohorts retained for context, including Meir’s older-adult population with mixed baseline activity, were explicitly labeled and excluded from estimates for the original induction target. Full-text exclusion decisions are listed in Supplementary Table S7.

### 2.3. Screening and Data Extraction

Records were managed in Zotero. Automated detection identified 168 duplicates, and manual checking identified 18 additional duplicates. Two reviewers independently screened titles and abstracts, followed by full-text assessment. Before formal screening, 23 records were classified as include, exclude, or uncertain to calibrate decisions. Agreement was summarized using unweighted Cohen’s κ. Disagreements were resolved through discussion or consultation with a third reviewer. No automated exclusion tool or artificial intelligence system was used for study screening. Calibration results are reported in Supplementary Table S2.

Two researchers independently extracted study design, population characteristics, treatment history, background medication, dietary protocols, outcome definitions, assessment times, and numerical results. Data were organized by study, outcome, time point, and analysis population. Conflicts were checked against the publication location corresponding to the relevant outcome and denominator. A further researcher checked the extraction, with disagreements resolved through discussion. Study authors were not contacted; uncertain information was excluded from quantitative inference. Shared centers, investigators, recruitment periods, and eligibility criteria were examined for possible overlap between reports.

Binary events were paired with their reported denominators, distinguishing randomized, modified intention-to-treat (ITT), baseline-active subgroup, and available-case populations. Treatment initiation, adherence, retention, and remission were recorded separately. Missing outcomes were not routinely imputed. Pasta’s published remission estimates retained the allocation-group denominators of 24 and 21. Five CDED participants withdrew because of intolerance within six weeks, so these proportions were interpreted in light of early attrition. No additional missing-outcome imputation was performed for Pasta. A conservative Szczubełek scenario treated two participants without week-6 assessment as non-remitters. Dileo’s ITT analysis classified non-adherent participants as treatment failures, and its full-cohort estimate was retained. Fliss-Isakov’s remission denominator comprised the 48 participants with documented baseline clinical activity; the activity and outcomes of all 152 counseled patients were incompletely characterized.

### 2.4. Outcomes and Data Harmonization

The primary outcome was clinical remission at week 6 during induction. Later assessments, usually week 12, were retained as separate secondary time-point analyses. Pasta met the broader study-level criteria for adult comparative evidence and contributed to the Mediterranean-diet (MD) stratum at weeks 12 and 24, with no week-6 primary-outcome data. Week-24 or later findings were classified as longer-term outcomes during treatment unless the source explicitly assessed follow-up after dietary cessation.

Clinical remission was defined by source-reported symptom thresholds, including HBI < 5 or CDAI < 150. Dileo’s HBI ≤ 4 is numerically equivalent to HBI < 5 for an integer score, although its analysis population differed; Sigall-Boneh’s stricter HBI ≤ 3 threshold was retained separately. Dileo additionally required the absence of systemic corticosteroids or budesonide for clinical remission. Biochemical and endoscopic remission required source-defined biomarker thresholds or endoscopic criteria, respectively. Reductions in CRP or FCP without a remission criterion were termed biochemical improvement. Sustained clinical remission required persistence from an earlier documented remission assessment; maintenance referred to preservation of previously achieved remission. Later cross-sectional remission and outcomes after dietary cessation were reported separately, as was clinical response.

Directly reported continuous statistics were preferred. Where harmonization was required, the Luo and Wan methods estimated means and standard deviations from reported summary statistics [21,22]. Original medians and interquartile ranges were retained for interpretation. Standard errors were converted using SD = SE × √n when the sample size was available. Graph-only values were extracted using WebPlotDigitizer, accessed on 2 June 2026. Ambiguous graph error bars were not used for inferential synthesis.

C-reactive protein (CRP) was expressed in mg/L and fecal calprotectin (FCP) in μg/g; mg/kg and μg/g are numerically equivalent. Key extraction and harmonization decisions are summarized in Supplementary Table S3. Supplementary Data S1 contains the complete binary and continuous extraction data, source locations, original statistics, and transformation records. No continuous outcome was pooled.

### 2.5. Risk of Bias and Certainty

Two researchers independently assessed randomized remission outcomes using RoB 2 [23]. Yanai was assessed at week 6. One set of Pasta judgments covered weeks 12 and 24 because outcome definitions, measurement, and domain considerations were shared. Domain judgments and supporting explanations are reported in Supplementary Table S4 and Figure S1. Outcome measurement, deviations from intended interventions, and missing-outcome handling were considered separately.

Uncontrolled studies were appraised using the JBI Critical Appraisal Checklist for Case Series [24]. Items were recorded as Yes, No, Unclear, or Not applicable, without an overall numerical score or categorical study-quality label (Supplementary Table S5; Figure S2). JBI appraisals describe methodological reporting in uncontrolled studies and were not translated into RoB 2 categories or causal efficacy ratings. Population applicability was assessed separately.

Comparative evidence certainty was assessed using GRADE, separately by comparator and time point [25]. Randomized evidence started at high certainty and was considered for downgrading across the standard domains. Inconsistency and publication bias could not be formally assessed with one trial per comparison; neither domain was automatically downgraded for this reason. Ratings apply to the broader trial populations, rather than a verified HBI 5–8 subgroup. Absolute effects were calculated as observed risk differences (RDs), with confidence intervals, and expressed per 1000 participants in Table 2, with the detailed assessment in Supplementary Table S6. Uncontrolled descriptive proportions were not assigned formal GRADE ratings.

### 2.6. Statistical Synthesis

Risk ratios (RRs), RDs, and 95% confidence intervals (CIs) were calculated from reported trial counts. RR intervals used the log-Wald method; RD intervals used binomial variances with a Wald approximation. All cells contained events, so no continuity correction was required. Yanai’s CDED + PEN versus CDED comparison at week 6 was analyzed separately from Pasta’s CDED versus MD comparisons at weeks 12 and 24. No overall comparative effect was calculated.

Uncontrolled remission proportions were reported individually with Clopper–Pearson exact binomial 95% CIs. Fliss-Isakov 2023 [17] reported 30/48 among participants with baseline HBI ≥5, and Szczubełek 2021 [18] reported 23/30 among those with baseline CDAI >150. Differences in activity indices, recruitment, background therapy, and missing-outcome handling precluded a common summary estimate. The conservative Szczubełek scenario used 23/32 and was reported separately.

Dileo’s week-6 ITT result (22/36) was presented separately because eligibility permitted objective disease activity without uniformly elevated baseline HBI. This full-cohort proportion describes remission status and does not estimate induction among a uniformly baseline-active clinical population. Sigall-Boneh’s adult result was retained separately because of its stricter HBI ≤3 threshold and treatment context. Uncontrolled proportions describe outcomes in the selected study populations; no indirect comparison with RCT effects or other diets was performed.

Trial-specific comparative calculations and study-level binomial intervals were executed in R 4.5.2 using the extracted event counts and the base-R implementation in Supplementary Code S1. No pooled uncontrolled estimate or heterogeneity statistic was reported in the revised synthesis. Meta-regression, subgroup meta-analysis, and statistical assessment of publication bias were not performed.

Evidence unsuitable for pooling was organized by design, comparator, time point, and outcome, retaining event/denominator pairs or original continuous summaries. Interpretation considered missingness, selection, applicability, and agreement between symptomatic and objective inflammatory outcomes, rather than the number of statistically significant results.

## 3. Results

### 3.1. Study Selection and Characteristics

**Figure 1:**
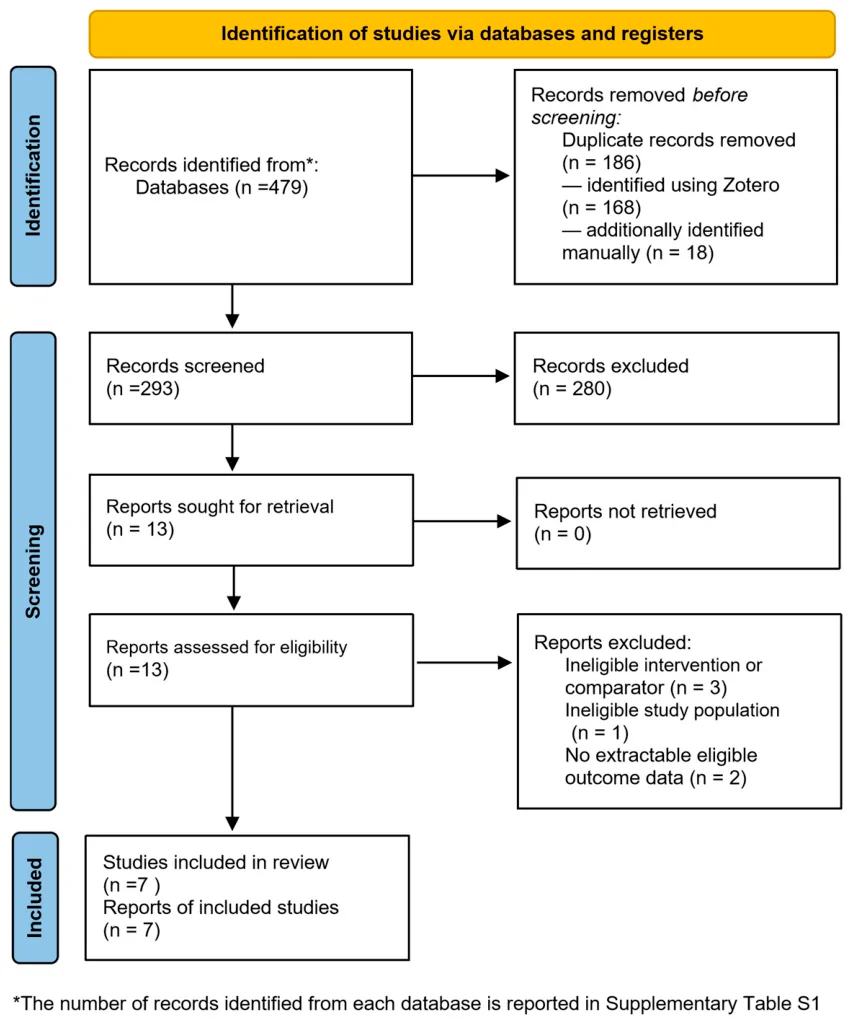
Study selection according to PRISMA 2020. Records were identified through six databases. Database-specific counts and full-text exclusion decisions are provided in Supplementary Tables S1 and S7, respectively.

The search identified 479 records. After removing 186 duplicates, 293 records underwent screening; 280 were excluded by title or abstract. Thirteen reports underwent full-text assessment, of which seven were included and six excluded (Figure 1; Supplementary Table S7). Pilot calibration agreement was 22/23 (95.7%), with κ = 0.783 (95% CI 0.375–1.000; Supplementary Table S2).

The evidence comprised two open-label RCTs and five uncontrolled studies published between 2014 and 2026 [15,16,17,18,19,20]. Sigall-Boneh contributed a separately extractable adult subgroup from a mixed-age report [13]. The trials contributed 85 analyzed participants. Yanai randomized 44 participants and analyzed 40 who received treatment for at least 48 h. The principal uncontrolled datasets comprised 48 baseline clinically active participants in Fliss-Isakov and 30 week-6 available cases in Szczubełek. Dileo contributed a full-cohort ITT remission-status estimate from 36 participants.

**Figure 2:**
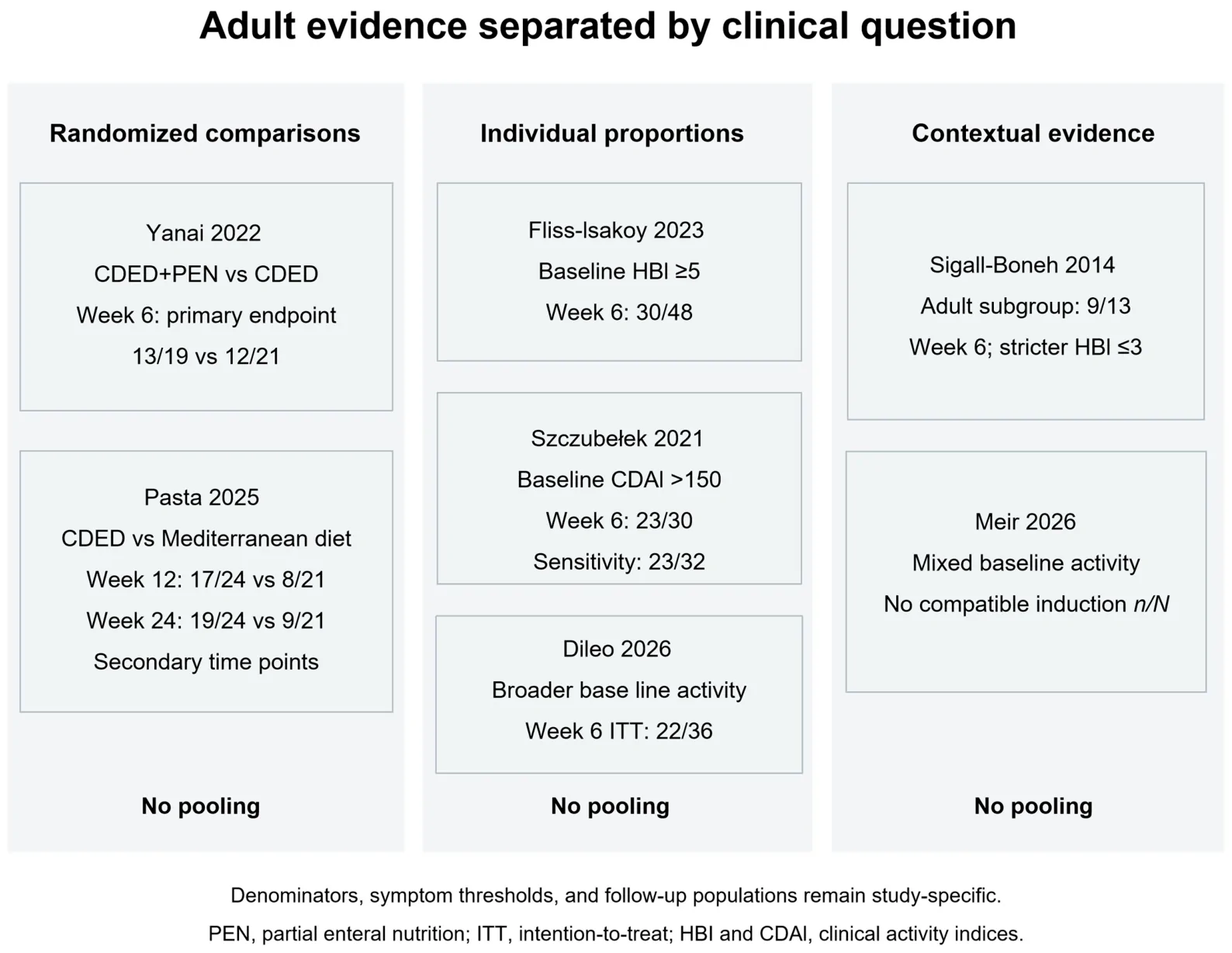
Evidence synthesis by clinical question and analysis population. Randomized comparisons are separated by comparator and time point. Uncontrolled studies are displayed individually without pooling; baseline activity definitions and analysis denominators remain study-specific. Dileo’s full-cohort estimate describes week-6 remission status in a population with mixed baseline clinical activity, rather than induction in a uniformly active population. Meir provides contextual evidence without a compatible induction event count. CDED, Crohn’s disease exclusion diet; PEN, partial enteral nutrition; ITT, intention-to-treat. Source studies: [13,15,16,17,18,19,20].

Disease duration, biologic exposure, background medication, and PEN delivery varied across studies (Table 1). The trials addressed distinct comparisons, and uncontrolled studies represented different analysis populations (Table 3; Figure 2). Fliss-Isakov and Meir reported patients from Tel Aviv Medical Center during 2018–2021 and January 2018–April 2024, respectively, creating potential overlap [17,19]. Yanai was a multicenter trial recruiting during 2017–2020 [15] and shared investigators with Fliss-Isakov. Its 18–55-year eligibility range differed from Meir’s >60-year criterion, but differences in entry criteria do not establish patient-level independence across calendar time. The reports were presented separately without a unique-patient total or an assumption of independent replication. Report-level provenance is summarized in Supplementary Table S11.

### 3.2. Methodological Appraisal

Both randomized remission comparisons were rated at overall high risk of bias, principally because HBI assessment was unblinded and included subjective components. Selective reporting raised some concerns because sufficiently detailed prespecified analysis information could not be confirmed. Post-randomization exclusions and missing-outcome handling also raised concerns in specific domains (Supplementary Table S4).

JBI appraisal rated all items Yes for Sigall-Boneh and Dileo. Fliss-Isakov received an Unclear rating for standardized and reliable measurement, and Szczubełek received Unclear ratings for consecutive and complete inclusion. Meir received No for appropriate statistical analysis because repeated observations and changing available-case denominators were not adequately addressed. Item-level findings are reported in Supplementary Table S5, without an overall study score.

### 3.3. Comparative Remission Outcomes

In Yanai, week-6 remission occurred in 13/19 participants receiving CDED + PEN and 12/21 receiving CDED alone. The RR was 1.20 (95% CI 0.74–1.94), and the RD was 11.3 percentage points (95% CI −18.5 to 41.0). The interval included effects in either direction, leaving the incremental benefit of PEN uncertain.

**Figure 3:**
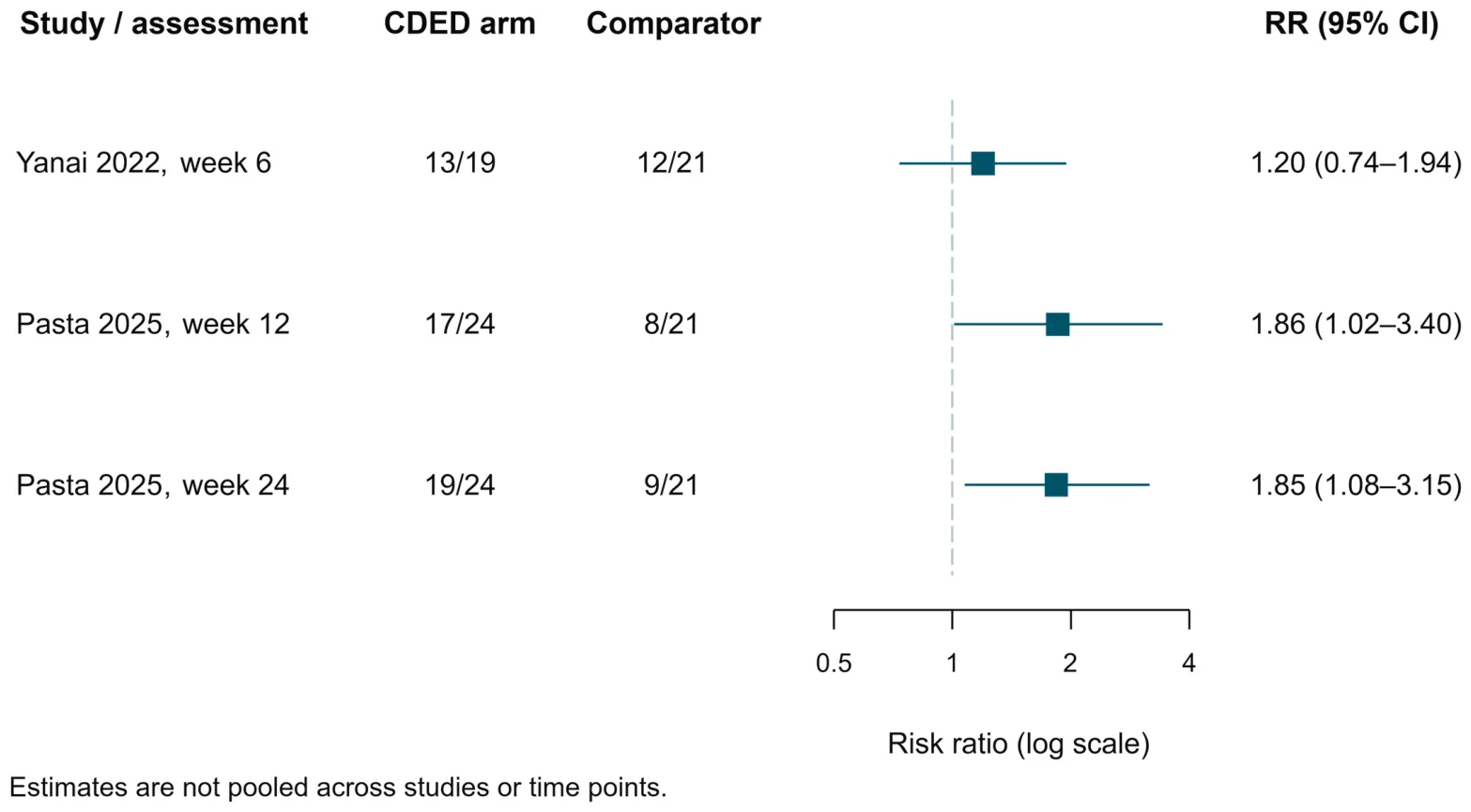
Trial-specific estimates of clinical remission. Squares indicate risk ratios (RRs), and horizontal lines indicate 95% log-Wald confidence intervals (CIs). The reference line is RR = 1. Yanai compares CDED + PEN with CDED alone at week 6; Pasta compares CDED with a Mediterranean diet at weeks 12 and 24. Effects are not pooled across trials or time points. CDED, Crohn’s disease exclusion diet; PEN, partial enteral nutrition. Source trials: [15,16].

In Pasta, week-12 remission occurred in 17/24 participants assigned CDED and 8/21 assigned MD, with an RR of 1.86 (95% CI 1.02–3.40). The RD was 32.7 percentage points (95% CI 5.1–60.3). At week 24, remission occurred in 19/24 versus 9/21 participants, with an RR of 1.85 (95% CI 1.08–3.15). The corresponding RD was 36.3 percentage points (95% CI 9.6–63.0). The published group denominators were retained, and results were interpreted in light of five early CDED withdrawals. This review performed no additional missing-outcome imputation. Both were secondary time-point analyses. Week-24 findings described remission during ongoing dietary treatment, with persistence from an earlier remission assessment and outcomes after cessation remaining separate questions (Figure 3).

Comparative certainty was very low for adding PEN at week 6 and low for CDED versus MD at weeks 12 and 24. Risk of bias and imprecision drove downgrading (Table 2; Supplementary Table S6). The symptomatic remission advantage in Pasta was not accompanied by a consistently demonstrated comparative advantage in CRP or FCP. Evidence for comparative endoscopic superiority remained insufficient.

### 3.4. Individual Uncontrolled Remission Outcomes and Denominator Sensitivity

**Figure 4:**
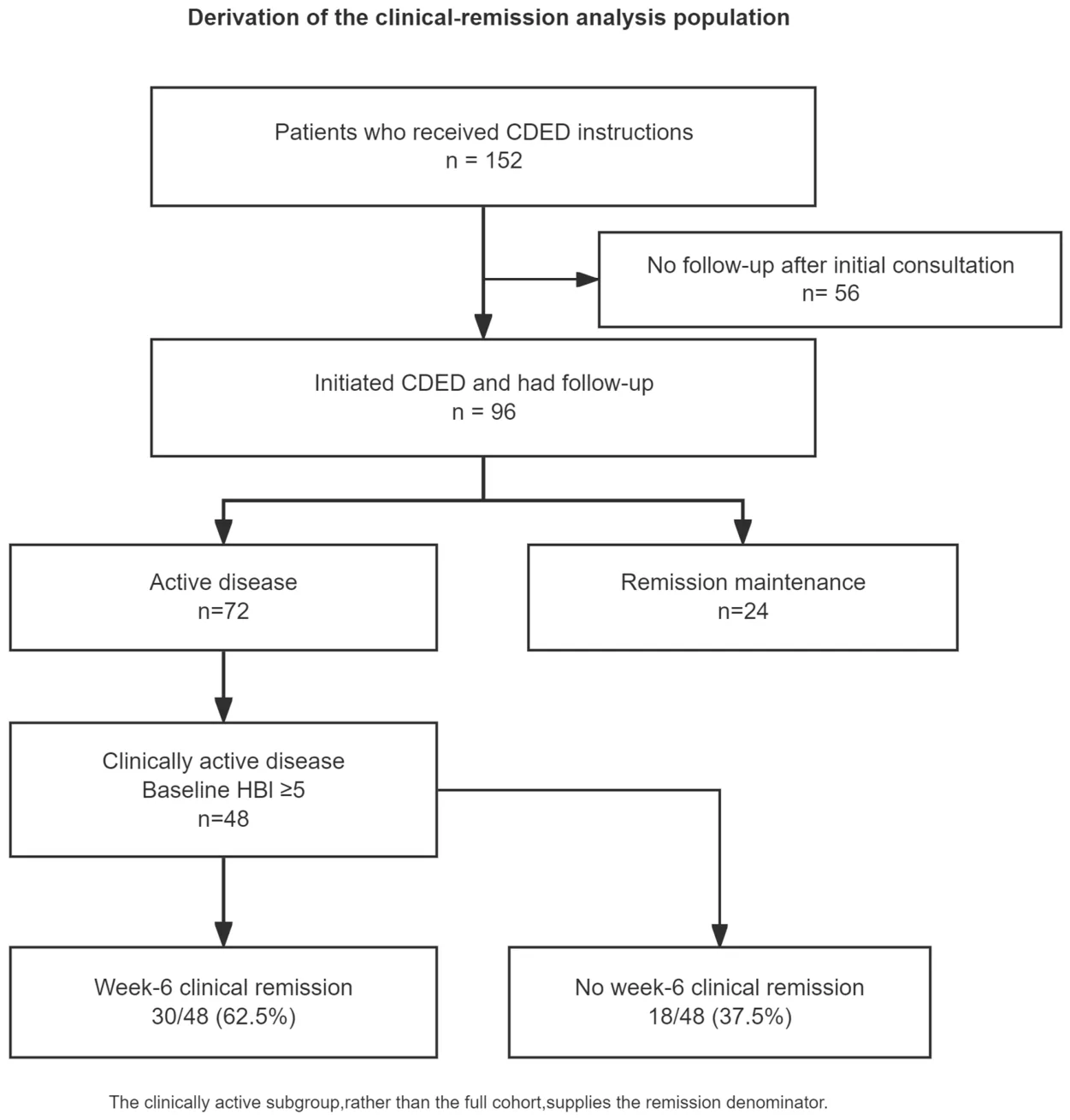
Derivation of the Fliss-Isakov clinical-remission population. Of 152 patients receiving CDED instructions, 96 had documented diet initiation with follow-up. Seventy-two were treated for active disease; the 48 with baseline HBI ≥5 supplied the clinical-remission denominator. Thirty achieved remission at week 6. CDED, Crohn’s disease exclusion diet; HBI, Harvey–Bradshaw Index. Source: Fliss-Isakov et al. [17].

In Fliss-Isakov, 152 patients received CDED instructions, 96 had documented treatment initiation with follow-up, and 72 were treated for active disease. Among 48 participants with baseline HBI ≥5, 30 achieved week-6 clinical remission (62.5%; exact 95% CI 47.4–76.0%; Figure 4). Szczubełek reported week-6 remission in 23/30 available cases (76.7%; 95% CI 57.7–90.1%) from 32 participants at baseline. These estimates retain their baseline-active and available-case denominators, respectively.

Treating the two participants without a Szczubełek week-6 assessment as non-remitters yielded 23/32 (71.9%; exact 95% CI 53.3–86.3%). This study-level sensitivity analysis shows the effect of the missing-outcome assumption while retaining the other features of the source population and treatment setting.

**Figure 5:**
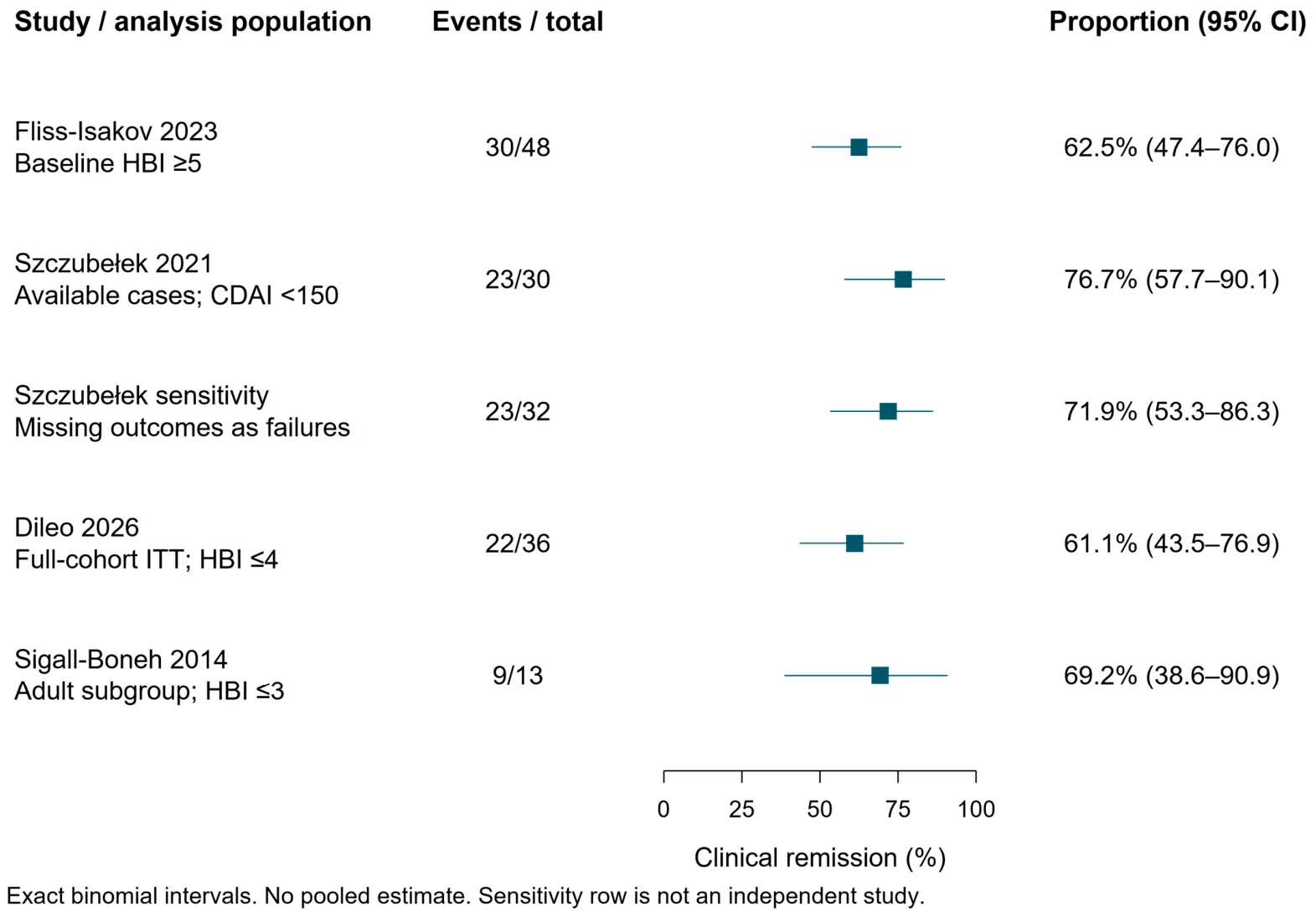
Individual week-6 clinical-remission proportions in uncontrolled studies. Squares and horizontal lines show study proportions and Clopper–Pearson exact binomial 95% confidence intervals. Baseline activity, analysis populations, and remission thresholds remain study-specific. Fliss-Isakov’s HBI ≥ 5 denotes baseline activity; Szczubełek’s CDAI < 150 denotes its remission threshold. The 23/32 Szczubełek sensitivity scenario counts two missing assessments as non-remission and represents an alternative denominator for the same study. Dileo uses a full-cohort ITT denominator with broader activity eligibility; Sigall-Boneh uses the stricter HBI ≤3 threshold. Estimates are descriptive and are displayed without pooling. Source studies: [13,17,18,20].

Dileo reported week-6 clinical remission in 22/36 participants using its ITT approach (61.1%; exact 95% CI 43.5–76.9%), compared with 22/22 adherent participants [20]. Non-adherent participants were classified as failures in the full-cohort analysis. The two denominators distinguish outcomes among all enrolled participants from those persisting with treatment. Baseline clinical activity was not required for every participant. Therefore, 22/36 describes remission status in the enrolled cohort and does not estimate induction among a uniformly baseline-active clinical population. Figure 5 presents the individual estimates alongside their analysis populations.

Sigall-Boneh’s adult subgroup achieved week-6 remission in 9/13 participants (69.2%) using HBI ≤ 3 [13]. Its stricter definition and treatment-experienced population were retained as separate evidence. Meir included 40 adults aged >60 years, with 24 in remission and 16 with active disease at baseline; a compatible induction event count was unavailable. In Dileo, 14/36 participants were in clinical remission at week 52 during ongoing treatment (38.9%), equivalent to 14/22 long-term adherent participants.

### 3.5. Clinical Response and Objective Inflammatory Outcomes

Clinical-response definitions and denominators varied. Yanai reported week-6 response, defined as an HBI reduction of ≥3 points, in 14/19 versus 14/21 participants. Fliss-Isakov reported week-12 response in 33/72, using a different denominator from its remission analysis. Szczubełek reported response in 25/30 at week 6 and 24/28 at week 12. Sigall-Boneh’s 37/47 response result described the entire mixed-age cohort and is reported only as context.

CRP and FCP were reported by seven and six studies, respectively, using different thresholds, summaries, and follow-up populations. In Yanai, median CRP changed from 15.8 to 7.4 mg/L with CDED + PEN and from 12.1 to 6.1 mg/L with CDED alone at week 12. Corresponding FCP medians changed from 229 to 104.1 μg/g and from 294.5 to 97.3 μg/g. These descriptive changes were based on biomarker samples smaller than the trial analysis groups.

In Pasta, median FCP was 273, 263, and 231 μg/g with CDED at baseline, week 12, and week 24, respectively. Corresponding values with MD were 200, 269, and 252 μg/g, and CRP showed no clear, consistent between-group pattern. Meir reported FCP changing from 444 to 321 μg/g at week 12 among evaluable participants with elevated baseline values. CRP changed from 11 to 10 mg/L among those with elevated baseline CRP. Small, changing denominators constrained interpretation of these longitudinal results.

Dileo’s longitudinal biomarker analysis included 22 adherent participants. Median FCP changed from 439.5 μg/g at baseline to 241.5, 238.5, and 243.5 μg/g at weeks 6, 12, and 52. Corresponding CRP medians were 1.0, 0.8, 0.5, and 0.7 mg/L. Source-defined biochemical remission required CRP ≤5 mg/L and FCP ≤ 250 μg/g without systemic corticosteroids or budesonide. It increased from 36.4% at week 6 to 50.0% at week 52 among adherent participants. Overall time effects for CRP and FCP were not statistically significant.

Yanai reported week-24 endoscopic remission in 8/19 versus 6/21 participants, using modified ITT denominators and classifying missing colonoscopy outcomes as non-remission. Sigall-Boneh reported mucosal healing or normal magnetic resonance enterography with FCP findings in 11/15 selected, reassessed patients from the mixed-age cohort. The latter findings provide mixed-age context. These endoscopic findings do not provide a CDED-versus-MD comparison. Across studies, symptomatic remission was more consistently reported than objective inflammatory control.

### 3.6. Adherence, Discontinuation, and Safety

Yanai reported high dietary adherence at week 6 in 12/19 participants receiving CDED + PEN and 18/21 receiving CDED alone. Early discontinuation before week 24 occurred in 3/19 and 8/21 participants, respectively. Reasons included worsening disease, loss to follow-up, and other clinical events, rather than dietary adverse reactions alone.

Pasta reported intolerance-related withdrawal in 5/24 CDED participants during the first six weeks. Body mass index decreased from 25.8 to 24.5 kg/m^2^, alongside changes in fat and lean compartments. The nutritional implications of weight loss depend on body composition, dietary intake, and baseline nutritional risk.

In Fliss-Isakov, 56/152 patients did not return after initial instruction; their treatment initiation status was not established. Among the 96 participants with documented initiation and follow-up, three could not tolerate the diet because of palatability. Szczubełek reported that 4/32 participants discontinued dietary restrictions before week 12. Sigall-Boneh reported incomplete adherence in 5/47 participants from the mixed-age cohort. Meir’s complete-adherence proportions were 17/27, 15/21, and 5/9 at weeks 6, 12, and 24, respectively, reflecting evaluable participants rather than full-cohort retention.

In Dileo, 22/36 participants completed the 52-week protocol; all 14 discontinuations occurred within six weeks. Reported reasons included nausea (3/14), bloating (1/14), increased fecal output (6/14), dietary restrictiveness (1/14), PEN acceptability (1/14), and practical difficulties (2/14). Among the 22 long-term adherent participants, no serious intervention-related adverse event was reported. Transient nausea and bloating occurred in 4/22 and 3/22 participants, respectively. These adverse-event findings describe completers, whereas early discontinuation reflects tolerability and treatment persistence in a different analysis population. Safety definitions, active surveillance, and available follow-up varied across studies.

## 4. Discussion

### 4.1. Interpretation of the Adult Evidence

The adult evidence addresses three distinct treatment questions: adding PEN to CDED, choosing CDED rather than another diet, and outcomes during uncontrolled dietary treatment. Separating these questions reveals a symptom-defined remission advantage over MD at week 12 in one small trial, alongside uncertainty about the incremental benefit of PEN at week 6. The corresponding evidence for objective inflammatory control was less consistent. Because HBI includes symptoms and well-being, symptomatic improvement is clinically relevant but may reflect both inflammatory and non-inflammatory changes, including expectations in an unblinded intervention. Joint assessment of clinical, biochemical, and endoscopic outcomes is therefore central to interpreting adult dietary treatment.

Analysis populations connect observed remission with the practical stages of dietary care. Fliss-Isakov’s clinically active subgroup, Dileo’s enrolled and adherent populations, and Szczubełek’s available cases represent different stages between counseling, initiation, persistence, and assessment. Reporting these stages separately clarifies both the outcomes achieved and the population to which they apply. Dileo’s full-cohort remission status cannot establish induction success specifically among participants with clinical activity at baseline. The uncontrolled estimates describe treatment experience; background therapy, selection, and incomplete follow-up remain alternative explanations for the observed outcomes.

Baseline activity and treatment history further define applicability. Trial populations extended beyond the initial HBI 5–8 target, and Dileo allowed objective activity without uniform clinical activity at baseline. Disease duration, biologic exposure, concomitant medication, and nutritional support also varied across studies. These characteristics locate each finding within its clinical setting; aggregate data do not establish their separate contributions to remission. Future comparative trials should align eligibility, objective disease activity, and the population used to estimate treatment outcomes.

### 4.2. Relation to Previous Reviews and Clinical Guidance

Comparator-specific presentation links the evidence to clinical decisions. Adding PEN to CDED at week 6 differs from choosing CDED rather than MD at weeks 12 or 24. Keeping the comparisons and treatment phases distinct makes each estimate interpretable for the corresponding nutritional strategy. In Pasta, nutritional-risk-based PEN was used in a subset of participants in both groups. Its comparison therefore represents dietary strategies with selective nutritional support and does not isolate the effect of CDED without PEN.

Earlier reviews provide complementary perspectives on dietary treatment (Supplementary Table S8). Correia reviewed pediatric and adult CDED studies without pooling, whereas Zhang evaluated multiple solid-food diets and excluded single-arm studies [9,11]. Batra searched through February 2025, included 17 studies, and reported an adult subgroup of four studies (134 participants; remission 64%, 95% CI 56–72%) [12]. The present search extends to 2 September 2026 and includes the later Meir and Dileo reports alongside overlapping core studies such as Yanai, Fliss-Isakov, and Szczubełek. Its contribution is the updated clinical interpretation of these data: comparator-specific RRs at distinct times, individual uncontrolled outcomes, and explicit links between remission, analysis populations, and objective inflammatory findings. Differences in study selection, denominators, and synthesis methods account for the distinct scope of these reviews.

ESPEN and AGA emphasize nutritional assessment and specialist dietetic support [1,2]. The 2025 ECCO dietary consensus states that CDED + PEN can be considered for induction in adults with mild-to-moderate CD; evidence for CDED alone or maintenance remains insufficient [26]. CDED + PEN and CDED alone represent distinct nutritional strategies, with different formula contributions and implementation requirements. Their use should be evaluated within an individualized treatment plan that assesses symptoms and objective inflammation together.

Dietary implementation also depends on dietitian availability, food access, cultural adaptation, and patients’ daily circumstances [10,27]. Nutritional monitoring should address unintended weight loss, nutrient adequacy, body composition, and the burden of restriction. These considerations connect remission outcomes with the feasibility and nutritional consequences of maintaining a restrictive diet.

### 4.3. Strengths and Limitations

The review links adult remission findings to clinically distinct comparisons, assessment times, and analysis populations. Individual presentation of uncontrolled outcomes preserves differences in eligibility and follow-up, while explicit event counts and denominators support interpretation. The extraction workbook retains original summary statistics and source locations to facilitate checking and reuse.

The principal limits on comparative inference are small samples, one trial per comparison, open-label symptom assessment, and heterogeneous background therapy. Selection between counseling, treatment initiation, and follow-up affects the population represented by each uncontrolled proportion. In Pasta, early CDED withdrawal further limits interpretation of remission estimates using the published allocation-group denominators. Possible overlap between Fliss-Isakov and Meir, and unresolved overlap between Yanai and Fliss-Isakov, also limit interpretation of the evidence volume. Reports were therefore kept separate without a combined unique-patient total; individual-level linkage would be needed to establish independence.

At the review level, the explicit departure from the initial activity-range target limits direct applicability to HBI 5–8 adults. Uncontrolled findings were reported individually, and the missing-outcome scenario remained exploratory. Adult- and design-related search terms may have missed poorly indexed or mixed-age reports. Study authors were not contacted to resolve overlap, uncertain subgroup data, or missing outcomes.

Some continuous data required conversion or graph digitization and were not pooled. Inconsistent assessment of inflammation and endoscopy limits conclusions about mucosal healing and durable inflammatory control, while nutritional adequacy and safety also require fuller characterization. Exact binomial intervals quantify sampling uncertainty within each study; selection, confounding, and differences in treatment setting require separate consideration.

### 4.4. Implications for Research

Adult trials should distinguish the incremental contribution of PEN from the comparative value of CDED against another clinically relevant diet. Clinical remission should be paired with objective inflammatory endpoints, stable background therapy, and blinded outcome assessment where feasible. Nutritional evaluation should quantify PEN energy contribution, total nutrient intake, body composition, unintended weight loss, and adherence by dietary phase. Participant flow should document counseling, enrollment, treatment initiation, assessment, adherence, and withdrawal, including reasons and timing. Longer-term assessment should distinguish remission during ongoing treatment, persistence among earlier responders, and relapse after dietary cessation. Together, these measures would connect disease control with nutritional adequacy and the feasibility of sustained dietary care.

## 5. Conclusions

This review links adult CDED remission outcomes to specific comparators, assessment times, and analysis populations. One trial suggests a symptomatic remission advantage over MD at week 12; the incremental contribution of PEN at week 6 remains uncertain. These findings are of low or very low certainty, and a consistent comparative advantage in biochemical or endoscopic remission has not been established. The clinical priority is to identify adults who achieve inflammatory control alongside symptom improvement, nutritional adequacy, and sustained dietary participation. Trials that assess these outcomes together will better define the role of CDED in adult care.

## Figures and Tables

**Table 1:** Study characteristics and dietary treatment context. Panel (**A**) Populations and analysis denominators. Panel (**B**) Background medication and dietary implementation.

(A)		
Study/Design	Population and Disease Context	Analysis Population/Treatment
Yanai 2022 [15]; open-label pilot RCT	18–55 years; biologic-naïve; HBI 5–14; uncomplicated CD; disease duration ≤ 5 years	44 randomized; 40 analyzed after ≥48 h treatment: CDED + PEN 19, CDED alone 21
Pasta 2025 [16]; open-label RCT	18–80 years; HBI 5–16; established background therapy permitted	CDED 24 versus Mediterranean diet 21
Fliss-Isakov 2023 [17]; retrospective cohort	Heterogeneous adult CD; clinical, biochemical, or endoscopic activity	152 instructed; 96 with documented initiation and follow-up; 72 active; 48 baseline HBI ≥ 5 for remission
Szczubełek 2021 [18]; prospective single-arm study	Adult active CD; CDAI > 150; stable maintenance medication permitted	32 baseline; 30 week-6 and 28 week-12 available cases; CDED + PEN
Sigall-Boneh 2014 [13]; prospective case series	34 children and 13 adults; treatment-experienced adult subgroup; HBI ≤ 3 remission	13 adults contribute 9/13 week-6 remission; mixed-age outcomes labeled separately
Meir 2026 [19]; retrospective cohort	Adults > 60 years; mixed active and remission populations	40 participants: 16 active and 24 in remission at baseline
Dileo 2026 [20]; prospective cohort	18–75 years; clinical plus objective activity, or active endoscopy/imaging; no uniform baseline HBI > 4 requirement	36 baseline; week-6 ITT remission status: 22/36; 22 long-term adherent participants
(**B**)		
**Study**	**Background Medication**	**Diet Phases and PEN Delivery**
Yanai 2022 [15]	Biologics, immunomodulators, and systemic corticosteroids excluded; limited stable mesalamine use	Weeks 0–6, 7–12, and 13–24. PEN arm: 1000 kcal/day initially, then 600 kcal/day; optional after week 12. Energy percentage NR.
Pasta 2025 [16]	Biologic/conventional immunosuppression initiated ≥24 weeks earlier; corticosteroids/antibiotics excluded	Weeks 1–6, 7–12, and 13–24. PEN 10–20% only for nutritional risk: 4/24 CDED and 2/21 control participants.
Fliss-Isakov 2023 [17]	Heterogeneous real-world treatment; no universal stability requirement	Phase-specific assessments at weeks 6, 12, and 24; some entered later phases. Individualized PEN; 21/48 clinically active participants received it.
Szczubełek 2021 [18]	5-ASA, immunomodulators, or biologics at fixed doses >8 weeks; steroids/antibiotics excluded	PEN 50% during weeks 0–6 and 25% during weeks 7–12; reduced/no formula permitted if refused; no structured CDED after week 12.
Sigall-Boneh 2014 [13]	Stable maintenance treatment permitted, including unchanged failed biologic treatment; induction therapies excluded	PEN up to 50%, maximum 1250 kcal/day, during weeks 0–6; 25% during weeks 7–12; optional formula when unable/unwilling.
Meir 2026 [19]	Concomitant real-world medication permitted; 8/40 receiving budesonide or prednisone at baseline	Most began Phase 1; some entered Phases 2/3. PEN recommended to 12/40 according to need; energy percentage NR.
Dileo 2026 [20]	Recent biologic/small-molecule changes and systemic corticosteroids excluded	PEN 50% during weeks 0–6, then 25% during weeks 7–52; structured three-phase CDED.

Abbreviations: 5-ASA, 5-aminosalicylic acid; CD, Crohn’s disease; CDAI, Crohn’s Disease Activity Index; CDED, Crohn’s disease exclusion diet; HBI, Harvey–Bradshaw Index; ITT, intention-to-treat; NR, not reported; PEN, partial enteral nutrition; RCT, randomized controlled trial. Full characteristics are provided in Supplementary Table S10.

**Table 2:** Summary of findings and GRADE certainty for randomized clinical-remission comparisons.

Comparison/Time	Clinical Remission CDED Arm/Comparator	RR (95% CI)	Absolute Difference per 1000 (95% CI)	Certainty	Main Concerns
CDED + PEN vs. CDED Week 6	13/19 vs. 12/21 1 RCT; *n* = 40	1.20 (0.74–1.94)	113 more (185 fewer to 410 more)	Very low	Risk of bias; imprecision
CDED vs. MD Week 12	17/24 vs. 8/21 1 RCT; *n* = 45	1.86 (1.02–3.40)	327 more (51 to 603 more)	Low	Risk of bias; imprecision
CDED vs. MD Week 24	19/24 vs. 9/21 1 RCT; *n* = 45	1.85 (1.08–3.15)	363 more (96 to 630 more)	Low	Risk of bias; imprecision

Absolute differences are observed risk differences with Wald 95% CIs, expressed per 1000. All outcomes are symptom-defined remission (HBI < 5) during treatment. Certainty was downgraded by one level for serious risk of bias in both trials and by two levels for very serious imprecision in Yanai or one level for serious imprecision in Pasta. With one trial per comparison, inconsistency and publication bias were unassessable and received no additional downgrade. Indirectness ratings concern the enrolled populations; applicability to HBI 5–8 remains unverified. Weeks 12 and 24 represent repeated assessments of the same Pasta trial, whereas Yanai addresses the week-6 primary endpoint. Pasta estimates retain the published allocation-group denominators and should be interpreted in light of early CDED withdrawal. Detailed justifications are provided in Supplementary Table S6.

**Table 3:** Outcome-specific synthesis decisions.

Study	Outcome-Specific Data	Role in Synthesis/Limitation
Yanai 2022 [15]	Week 6: 13/19 vs. 12/21	Separate comparative effect; modified ITT population
Pasta 2025 [16]	Week 12: 17/24 vs. 8/21; week 24: 19/24 vs. 9/21	Separate secondary time-point comparisons; no week-6 primary-outcome data; published group denominators retained despite early withdrawal
Fliss-Isakov 2023 [17]	Week 6: 30/48	Individual descriptive estimate; selected baseline clinically active subgroup
Szczubełek 2021 [18]	Week 6: 23/30; conservative 23/32	Individual descriptive estimate; missing-as-failure sensitivity shown separately
Dileo 2026 [20]	Week 6 ITT remission status: 22/36; week 52 remission: 14/36	Descriptive remission status; mixed baseline clinical activity; no induction-only estimate or pooling
Sigall-Boneh 2014 [13]	Adult subgroup week 6: 9/13	Separate narrative estimate; stricter HBI ≤ 3 and treatment context
Meir 2026 [19]	Continuous outcomes and adherence; no compatible induction *n/N*	Narrative; baseline includes remission and follow-up denominators change

Estimates retain the outcome-specific analysis populations of the source studies; no indirect comparison with randomized trial effects was performed.

## Data Availability

The analyzed data were extracted from published reports. Study-level data and harmonization records are provided in Supplementary Data S1, and executable aggregate-data analyses are provided in Supplementary Code S1. Search strategies and full-text exclusion decisions are reported in Supplementary Tables S1 and S7. No original participant-level data were collected.

## Supplementary Materials

The following supporting information can be downloaded at: https://www.mdpi.com/article/10.3390/nu18213656/s1, Table S1: Search strategies for all databases; Table S2: Pilot calibration of inter-reviewer agreement before formal title and abstract screening; Table S3: Key data-extraction and harmonization decisions; Table S4: Risk-of-bias judgments for clinical remission in the included randomized controlled trials, assessed using the Cochrane Risk of Bias 2 (RoB 2) tool; Table S5: JBI critical appraisal of the included single-arm studies using the JBI Critical Appraisal Checklist for Case Series; Table S6: GRADE certainty of evidence for comparative clinical remission outcomes; Table S7: Full-text reports excluded after eligibility assessment and reasons for exclusion; Table S8: Comparison of the present review with previous systematic reviews of dietary therapy and CDED in Crohn’s disease; Table S9: Individual uncontrolled week-6 remission estimates and denominator sensitivity; Table S10: Full study characteristics and treatment-context fields; Table S11: Report-level cohort provenance and possible patient overlap; Figure S1: Traffic-light plot of risk-of-bias judgments for clinical remission in the included randomized controlled trials, assessed using RoB 2; Figure S2: Traffic-light plot of methodological appraisal judgments for the included single-arm studies, assessed using the JBI Critical Appraisal Checklist for Case Series; Supplementary Data S1: Study-level extraction and harmonization dataset; Supplementary Code S1: Aggregate-data analyses; and the PRISMA 2020 checklist. References cited in the supplementary files are included in the main reference list [8,9,11,12,13,14,15,16,17,18,19,20,21,22,23,24,25,27,28,29,30,31].

## Abbreviations

BMI	Body mass index
CD	Crohn’s disease
CDAI	Crohn’s Disease Activity Index
CDED	Crohn’s disease exclusion diet
CI	Confidence interval
CRP	C-reactive protein
EEN	Exclusive enteral nutrition
FCP	Fecal calprotectin
GRADE	Grading of Recommendations Assessment, Development and Evaluation
HBI	Harvey–Bradshaw Index
IBD	Inflammatory bowel disease
ITT	Intention-to-treat
JBI	Joanna Briggs Institute
MD	Mediterranean diet
NR	Not reported
PEN	Partial enteral nutrition
PRISMA	Preferred Reporting Items for Systematic Reviews and Meta-Analyses
RCT	Randomized controlled trial
RD	Risk difference
RoB 2	Risk of Bias 2
RR	Risk ratio
SD	Standard deviation
SE	Standard error
