# Supplementary material for: Crohn’s Disease Exclusion Diet for Remission Induction in Adults: A Systematic Review and Stratified Synthesis
Source: Nutrients. 2026 Nov 3;18(21):3656. doi: 10.3390/nu18213656 (PMC13765171; doi:10.3390/nu18213656)
Supplement: Supplementary file 1 [file nutrients-18-03656-s001.zip › Supplementary_Materials.pdf]

Supplementary Materials

Crohn’s Disease Exclusion Diet for Remission Induction in Adults: A Systematic Review and Stratified Synthesis

Supplementary Tables S1–S11 and Figures S1–S2. The extraction workbook, executable code, and PRISMA checklist are supplied separately. Bracketed reference numbers correspond to the reference list in the main manuscript.

Supplementary Table S1. Search strategies for all databases

PubMed

Platform: National Library of Medicine
Search date: 2 September 2026
Search strategy: ("Crohn Disease"[MeSH] OR "Crohn's Disease"[tiab] OR "regional enteritis"[tiab]) AND ("Crohn's Disease Exclusion Diet"[tiab] OR "CDED"[tiab] OR "exclusion diet"[tiab] OR "Levine diet"[tiab]) AND ("adult"[MeSH] OR "adults"[tiab]) AND ("clinical trial"[pt] OR "randomized"[tiab] OR "randomised"[tiab] OR "cohort"[tiab] OR "prospective"[tiab] OR "retrospective"[tiab] OR "observational"[tiab])
Filters applied: Publication date from 1 January 2014 to 2 September 2026; English and Chinese
Records retrieved: n = 20

Web of Science (All Databases)

Platform: Web of Science, Clarivate
Search date: 2 September 2026

Search strategy:
TS=("Crohn$ disease" OR "regional enteritis")
AND TS=("CDED" OR "Crohn$ disease exclusion diet" OR "exclusion diet" OR "Levine diet")
AND TS=("adult$" OR "aged")
AND TS=("clinical trial" OR "randomized" OR "cohort" OR "observational")

Timespan: 2014–2026
Records retrieved: n = 30

Cochrane Library

Platform: Cochrane
Search date: 2 September 2026

Search strategy:

MeSH descriptor [Crohn Disease] explode all trees
AND (CDED OR "Crohn$ disease exclusion diet" OR "exclusion diet" OR "Levine diet"):ti,ab,kw
AND (adult OR adults):ti,ab,kw [Year: 2014-2026]

Publication year: 2014–2026
Records retrieved: n = 16

Embase

Platform: Ovid
Search date: 2 September 2026

Search strategy:
1. exp Crohn disease/ OR (Crohn$ disease OR regional enteritis).ti,ab,kw.
2. (CDED OR "Crohn$ disease exclusion diet" OR "exclusion diet" OR "Levine diet").ti,ab,kw.
3. exp adult/ OR (adult$1 OR aged).ti,ab,kw.
4. exp clinical trial/ OR exp cohort analysis/ OR exp prospective study/ OR exp retrospective study/
5. 1 AND 2 AND 3 AND 4

Limits applied: Publication years 2014–2026; English or Chinese; humans
Records retrieved: n = 125

Scopus

Platform: Elsevier
Search date: 2 September 2026

Search strategy:
(TITLE-ABS-KEY("Crohn$ disease") AND TITLE-ABS-KEY(CDED OR "exclusion diet" OR "Levine diet") AND TITLE-ABS-KEY(adult OR aged)) AND PUBYEAR > 2013 AND PUBYEAR < 2027

Publication year: 2014–2026
Records retrieved: n = 111

China National Knowledge Infrastructure (CNKI)

Platform: China National Knowledge Infrastructure (CNKI), Advanced Search interface
Search date: 2 September 2026

Search field: Subject (主题)

Matching mode: Fuzzy matching
Search strategy:

主题 = (克罗恩病 + 克隆病 + 克隆氏病 + Crohn + "Crohn's disease")

AND

主题 = (CDED + "Crohn's Disease Exclusion Diet" + 克罗恩病排除饮食 + 排除饮食 + 排除性饮食 + Levine 饮食)

Publication date: 1 January 2014 to 2 September 2026
Records retrieved: n = 177
Export format: EndNote (.enw) format; records were imported into Zotero for deduplication and screening.

Note: All database searches were rerun on 2 September 2026. The record counts shown represent the final results of the full updated searches and total 479 records before deduplication.

Supplementary Table S2. Pilot calibration of inter-reviewer agreement before formal title and abstract screening

Panel A. Pilot screening cross-tabulation

Reviewer 1 / Reviewer 2	Include	Exclude	Uncertain	Total
Include	1	1	0	2
Exclude	0	20	0	20
Uncertain	0	0	1	1
Total	1	21	1	23

Panel B. Agreement statistics

Statistic	Result
Exact agreement	22/23(95.7%)
Expected agreement	80.00%
Cohen's κ	0.783
Standard error	0.208
95% CI	0.375–1.000

Note:A random sample of 23 records was used solely for pilot calibration of the screening criteria before formal title and abstract screening. These records were not intended to estimate inter-reviewer agreement across the entire formal screening stage. Screening decisions were classified as include, exclude, or uncertain. Cohen's κ was calculated without weighting because the categories were treated as nominal. Disagreements were resolved through discussion after independent assessment of the pilot records. CI, confidence interval.

Supplementary Table S3. Key data-extraction and harmonization decisions

Panel A. Key decisions for dichotomous outcomes and analysis denominators

Study / outcome	Data-extraction issue	Final decision	Implication for synthesis
Yanai et al., 2022 [15] — Week-6 clinical remission	Clinical response and clinical remission were reported separately and could be confused during extraction.	Week-6 clinical remission was 13/19 in the CDED+PEN group and 12/21 in the CDED-alone group (25/40 overall). The value 28/40 represented clinical response, not remission.	The published modified ITT analysis denominators (19 and 21), not all 44 randomized participants, were retained.
Yanai et al., 2022 [15] — Week-24 endoscopic remission	Modified ITT and complete-case colonoscopy denominators were available.	The published modified ITT estimates, 8/19 vs 6/21, were retained. Complete-case values 8/15 vs 6/13 were not substituted; the trial randomized 44 participants.	Retained as a secondary endoscopic outcome; complete-case denominators were not used for the principal estimate.
Yanai et al., 2022 [15] — early study discontinuation	Early discontinuation included several causes and should not be interpreted as equivalent to dietary intolerance.	All-cause early discontinuation was 3/19 for CDED+PEN and 8/21 for CDED alone. Disease worsening accounted for only part of these events.	Used narratively for feasibility and retention; not interpreted as a diet-related adverse-event rate.
Pasta et al., 2025 [16] — clinical remission	No Week-6 remission result was reported.	No Week-6 value was imputed. Clinical remission was extracted at Week 12 (17/24 vs 8/21) and Week 24 (19/24 vs 9/21).	Week-12 and Week-24 comparative estimates were analyzed separately and were not pooled with the Week-6 Yanai comparison.
Fliss-Isakov et al., 2023 [17] — clinical remission	The broader active-disease cohort comprised 72 participants, whereas remission was evaluated in a clinically active subgroup.	The remission denominator was restricted to participants with baseline HBI ≥5 (30/48 at Week 6). The broader n=72 cohort used for clinical response was not substituted.	30/48 is reported as an individual descriptive estimate for the baseline clinically active subgroup. Potential selection bias is considered in interpretation.

Study / outcome	Data-extraction issue	Final decision	Implication for synthesis
Szczubełek et al., 2021 [18] — clinical remission	Follow-up denominators differed from the baseline cohort of n=32.	Clinical remission was 23/30 at Week 6 and 23/28 at Week 12, using the participants assessed at the corresponding time point.	23/30 is reported individually. A post hoc same-study scenario treats the two missing week-6 assessments as non-remitters (23/32); the rows are not independent cohorts.
Sigall-Boneh et al., 2014 [13] — adult subgroup remission	The original study included a mixed-age cohort; the extractable adult subgroup was small and used a stricter remission definition.	The adult subgroup result of 9/13 was retained separately; remission was defined as HBI ≤3.	Used as supplementary narrative evidence only and not pooled with adult studies defining remission as HBI <5.
Meir et al., 2026 [19] — Week-6 induction remission	HBI changes were reported, but a compatible binary remission numerator and denominator were not available.	No binary remission event/total was reconstructed or imputed.	Excluded from quantitative remission synthesis; clinical, biomarker, adherence, and safety findings were summarized narratively.
Dileo et al., 2026 [20] — Week-6 clinical remission	Both ITT and adherent-population results were reported.	Week-6 ITT remission was 22/36; the adherent-population result was 22/22.	Individual descriptive ITT remission estimate; broader baseline-activity population, not pooled.

Panel B. Key decisions for continuous-outcome harmonization

Data issue	Final handling	Rationale / interpretation
Directly reported mean±SD vs converted estimates	Directly reported mean±SD values were prioritized whenever both directly reported and convertible summary statistics were available.	Avoided unnecessary transformation and preserved the original reported statistic where possible.
Median and IQR data	When conversion was required and the necessary sample size was available, the mean was estimated using the Luo et al. [21] method and the SD using the Wan et al. [22] method. Original median and IQR values were retained.	Converted mean±SD values were treated as approximations and did not replace the original reported median/IQR for narrative interpretation.
Mean±SE reported in figures	When mean and SE were available only graphically, values were digitized and SE was converted using $SD = SE \times \sqrt{n}$.	Allowed consistent expression of dispersion while retaining the graphical source and extraction method in the full dataset.
CRP units	CRP was standardized to mg/L; values reported in mg/dL were multiplied by 10.	Ensured consistent units across studies.

Data issue	Final handling	Rationale / interpretation
Fecal calprotectin units	Fecal calprotectin was standardized to µg/g; values reported as mg/kg were treated as numerically equivalent.	Ensured consistent units across studies without altering the numerical value.
Pasta et al., 2025 [16] — HBI error bars	Text-reported median/IQR values were prioritized. Legacy graph-derived values are retained in the extraction workbook for traceability only; the baseline graphical limits are not treated as IQRs in the revised interpretation or inferential synthesis.	The figure labels the error bars as 95% CIs. Their interpretation could not be reconciled sufficiently for inference. Legacy conversions remain identifiable in the extraction record but were not used in the revised statistical synthesis.
Szczubełek et al., 2021 [18] — baseline CDAI and fecal calprotectin	Directly reported values of CDAI 290.79±127.04 and fecal calprotectin 693.56±940.49 µg/g were retained.	Direct mean±SD values in the source table were prioritized over values that could otherwise have been estimated from median/IQR.
Very small or unreported follow-up samples	No conversion was performed when a meaningful summary statistic could not be derived, such as n=1 or when the follow-up sample size was not reported. Estimates based on very small available-case samples were interpreted narratively.	Avoided creating unstable or uninterpretable quantitative estimates from insufficiently reported data.

Panel C. Cross-cutting data-harmonization and synthesis rules

Harmonization issue	Prespecified / final handling	Purpose
Unit of data management	Data were managed at the study–outcome–time point–analysis population level.	Prevented repeated counting of different time points, subgroups, or analysis populations from the same study.
Conflicting information across report locations	When values differed across the main text, tables, figures, or supplementary materials, the source most directly corresponding to the prespecified outcome and analysis population was prioritized.	Ensured that the extracted numerator, denominator, and outcome definition referred to the same estimand.
Unverifiable or ambiguous data	Information that could not be confirmed from publicly available study materials was classified as unclear and was not used for quantitative inference. Study authors were not contacted for additional verification.	Avoided reconstructing or assuming unreported outcome data.

Harmonization issue	Prespecified / final handling	Purpose
Analysis-denominator selection	Denominators were matched to the population used for the specific outcome and time point. Broader cohorts, narrower subgroups, randomized denominators, and available-case denominators were not substituted for one another unless explicitly justified by the original study analysis.	Minimized denominator misclassification and inappropriate mixing of different analysis populations.
Outcome-definition harmonization	Outcomes with materially different definitions were retained separately. In particular, remission defined as HBI ≤3 was not treated as directly interchangeable with HBI <5 for the principal quantitative synthesis.	Preserved clinically relevant differences in outcome definitions.
Time-point and comparator harmonization	Comparative effects were retained separately by comparator and assessment time point; clinically distinct RCT comparisons were not pooled across studies.	Prevented pooling of different clinical questions and treatment phases.
Missing outcome data	Missing outcomes were not routinely imputed. The post hoc Szczubełek study-level scenario used 23/32; Dileo's reported ITT 22/36 was presented individually.	Assessed the numerical effect of this specific denominator assumption without claiming to resolve other sources of selection bias.
Eligibility for quantitative synthesis	Trial effects and individual uncontrolled proportions use reported adult analysis populations. Broader activity ranges are explicit, and uncontrolled studies are not pooled.	Prevented forced quantitative combination of non-comparable evidence.
Multiple reports or analyses from the same study population	Related outcomes, time points, subgroups, and companion analyses were mapped to the underlying study population and were not counted as independent participants or independent studies.	Prevented duplicate counting.

Abbreviations: CDED, Crohn's disease exclusion diet; PEN, partial enteral nutrition; HBI, Harvey–Bradshaw Index; CDAI, Crohn's Disease Activity Index; CRP, C-reactive protein; FCP, fecal calprotectin; IBDQ, Inflammatory Bowel Disease Questionnaire; ITT, intention-to-treat; IQR, interquartile range; SD, standard deviation; SE, standard error; CI, confidence interval.

This table documents extraction, analysis-population and harmonization decisions. Supplementary Data S1 retains original summaries, source locations and correction records. No unreported event count was reconstructed. The post hoc Szczubełek denominator scenario is explicitly distinguished from the reported available-case result.

Supplementary Table S4. Risk-of-bias judgments for the clinical remission outcome in the included randomized controlled trials, assessed using the Cochrane Risk of Bias 2 (RoB 2) tool [23].

Panel A. Domain-level risk-of-bias judgments

Study	Outcome and time point	D1	D2	D3	D4	D5	Overall
Pasta et al., 2025 [16]	Clinical remission assessed using HBI <5, Weeks 12 and 24	Low	Low	Some concerns	High	Some concerns	High
Yanai et al., 2022 [15]	Clinical remission assessed using HBI <5, Week 6	Low	Some concerns	Some concerns	High	Some concerns	High

Domain definitions: D1, bias arising from the randomization process; D2, bias due to deviations from intended interventions; D3, bias due to missing outcome data; D4, bias in measurement of the outcome; D5, bias in selection of the reported result.

Panel B. Support for judgment

Study	Domain	Judgment	Support for judgment
Pasta et al., 2025 [16]	D1: Bias arising from the randomization process	Low	The study was reported as randomized, and the available baseline characteristics did not indicate a material imbalance suggesting a problem with the randomization process. No evidence was identified that the allocation sequence was predictably generated or improperly implemented.
Pasta et al., 2025	D2: Bias due to deviations from intended interventions	Low	Blinding of participants and personnel was not feasible because of the nature of the dietary interventions. However, awareness of the assigned diet does not itself indicate bias due to deviations from intended intervention. No clear evidence of important trial-context–related deviations, differential co-interventions, or an analysis inconsistent with the assigned intervention groups was identified.
Pasta et al., 2025	D3: Bias due to missing outcome data	Some concerns	Five of 24 CDED participants withdrew because of intolerance during the first six weeks. Later proportions used allocated-group denominators, but this alone does not document how missing outcomes were obtained or assigned. The low-risk judgment was therefore not adequately supported.
Pasta et al., 2025	D4: Bias in measurement of the outcome	High	The trial was open label, and clinical remission was defined using HBI <5. HBI includes symptom-based and partly subjective components that may be influenced by participants' and investigators' knowledge of the assigned dietary intervention. Because such knowledge could plausibly influence symptom reporting or clinical assessment, the outcome measurement was considered likely to have been affected by lack of blinding.

Study	Domain	Judgment	Support for judgment
Pasta et al., 2025	D5: Bias in selection of the reported result	Some concerns	Clinical remission was reported at multiple follow-up time points, but the available protocol or prespecified analysis information was not sufficiently detailed to exclude selection among multiple eligible outcome measurements, time points, or analytic approaches. No direct evidence of selective reporting was identified, but prespecification could not be fully verified.
Pasta et al., 2025	Overall risk of bias	High	Overall high risk remains determined by outcome measurement. Missing-outcome handling also raises some concerns; the same revised profile is used for weeks 12 and 24.
Yanai et al., 2022 [15]	D1: Bias arising from the randomization process	Low	The study was reported as randomized, and the available baseline information did not show a substantial imbalance suggesting failure of the randomization process. No evidence was identified that the sequence generation or allocation process introduced systematic differences between groups.
Yanai et al., 2022	D2: Bias due to deviations from intended interventions	Some concerns	The published analysis excluded four of 44 randomized participants and retained 40 receiving intervention for at least 48 hours. This post-randomization restriction may depart from an assignment-effect analysis; a complete justification excluding bias was not established.
Yanai et al., 2022	D3: Bias due to missing outcome data	Some concerns	Forty of 44 randomized participants entered the published analysis (19 plus 21). These are not the complete randomized-group denominators. An unchanged denominator within this selected population is insufficient to establish absence of missing-data bias.
Yanai et al., 2022	D4: Bias in measurement of the outcome	High	The study was open label, and clinical remission was defined using HBI <5. Because HBI contains subjective symptom-based components, knowledge of the assigned intervention could plausibly influence participant reporting or investigator assessment. The absence of blinded outcome assessment was therefore considered likely to affect measurement of the clinical remission outcome.
Yanai et al., 2022	D5: Bias in selection of the reported result	Some concerns	Although Week-6 clinical remission was identified as a principal clinical outcome, the available protocol or prespecified statistical information was not sufficiently detailed to fully verify selection among multiple eligible measurements or analyses. There was no definite evidence of selective reporting, but the possibility could not be excluded.
Yanai et al., 2022	Overall risk of bias	High	The overall judgment was high because the clinical remission outcome was judged to be at high risk of bias in the outcome-measurement domain.

Abbreviations: CDED, Crohn’s disease exclusion diet; HBI, Harvey–Bradshaw Index; PEN, partial enteral nutrition; RoB 2, Risk of Bias 2.

Note: The RoB 2 assessment was conducted for the specified clinical remission results rather than as a general quality rating of the entire article. For Pasta et al., one set of judgments was applied to the Week-12 and Week-24 clinical remission results because the outcome definition, measurement approach, analysis population and resulting domain-level judgments were considered consistent across these time points.

Supplementary Figure S1:Traffic-light plot of risk-of-bias judgments for the clinical remission outcome in the included randomized controlled trials, assessed using the Cochrane Risk of Bias 2 (RoB 2) tool.

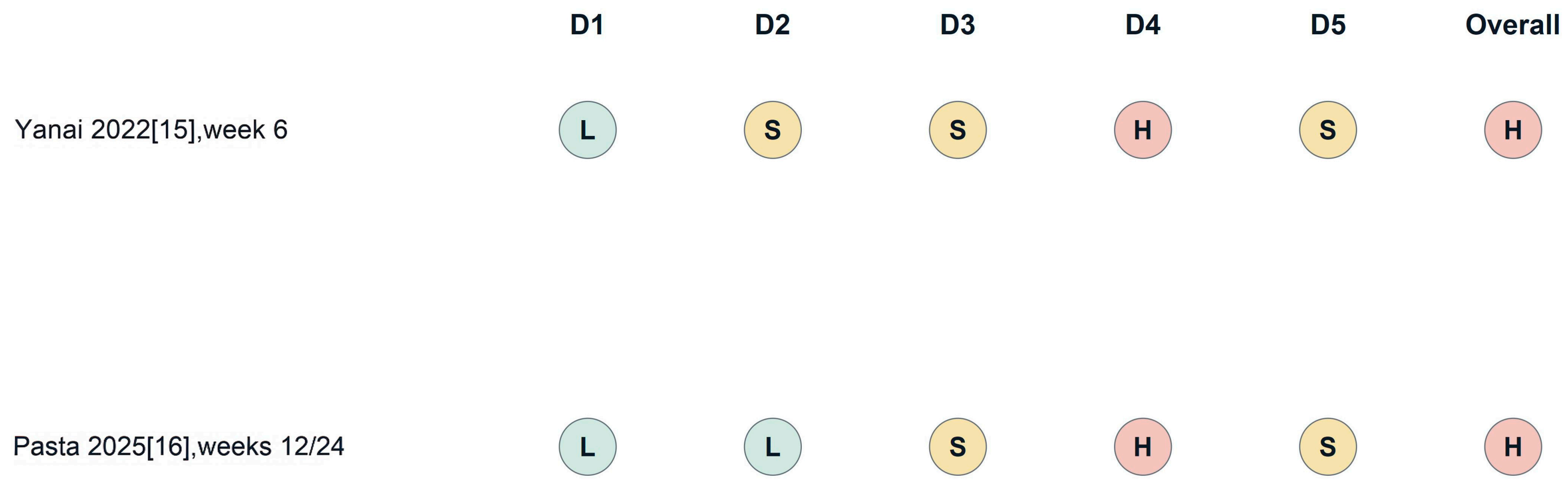

L, low risk; S, some concerns; H, high risk. Domains D1-D5 follow RoB 2.

Missing-data judgments revised during submission preparation; see supporting reasons in Table S4.

For Pasta, the same domain judgments apply at weeks 12 and 24. Yanai D2/D3 and Pasta D3 were revised during submission preparation; the overall high-risk judgments were unchanged.

Supplementary Table S5. JBI critical appraisal of the included single-arm studies using the JBI Critical Appraisal Checklist for Case Series [24].

Panel A. Item-level critical appraisal judgments

Study	Year	Q1	Q2	Q3	Q4	Q5	Q6	Q7	Q8	Q9	Q10
Szczubełek et al. [18]	2021	Y	Y	Y	U	U	Y	Y	Y	Y	Y
Sigall-Boneh et al. [13]	2014	Y	Y	Y	Y	Y	Y	Y	Y	Y	Y
Meir et al. [19]	2026	Y	Y	Y	Y	Y	Y	Y	Y	Y	N
Fliss-Isakov et al. [17]	2023	Y	U	Y	Y	Y	Y	Y	Y	Y	Y
Dileo et al. [20]	2026	Y	Y	Y	Y	Y	Y	Y	Y	Y	Y

Abbreviations: Y, Yes; N, No; U, Unclear; NA, Not applicable.

Panel B. Support for key judgments

Study	Item(s)	Judgment	Support for judgment
Szczubełek et al., 2021 [18]	Q1–Q3	Yes	The study reported defined eligibility criteria, identified participants as having Crohn disease using clinically accepted diagnostic procedures, and used established disease-activity and inflammatory measures.
Szczubełek et al., 2021	Q4	Unclear	The report did not clearly state that participants were enrolled consecutively during the recruitment period. The absence of an explicit consecutive-enrolment statement prevented a definitive “Yes” judgment.
Szczubełek et al., 2021	Q5	Unclear	It was not clearly reported whether all eligible patients presenting during the recruitment period were included or whether potentially eligible patients were omitted. Complete inclusion could therefore not be confirmed.
Szczubełek et al., 2021	Q6–Q9	Yes	Participant demographics, relevant clinical characteristics, follow-up outcomes and the clinical setting were adequately described for interpretation of the case series.
Szczubełek et al., 2021	Q10	Yes	The statistical analyses used to summarize the reported outcomes were considered appropriate for the study design and the form of the available data.
Sigall-Boneh et al., 2014 [13]	Q1–Q10	Yes	The study provided identifiable eligibility criteria, standardized identification and assessment of Crohn disease, consecutive and sufficiently complete inclusion, and adequate reporting of participant characteristics, clinical information, follow-up outcomes, study setting and statistical analyses. The mixed-age population and the characteristics of the extractable adult subgroup affect applicability to the review question but do not, by themselves, constitute a negative JBI case-series judgment.

Study	Item(s)	Judgment	Support for judgment
Meir et al., 2026 [19]	Q1–Q9	Yes	The study described its eligibility criteria, clinical identification of Crohn disease, participant inclusion, demographic and clinical characteristics, follow-up outcomes and study setting sufficiently for methodological appraisal.
Meir et al., 2026	Q10	No	The longitudinal analyses did not adequately account for the within-participant correlation arising from repeated measurements over time. In addition, the number of participants contributing data decreased substantially across follow-up time points, and the reported analyses did not fully address the implications of the changing available-case denominators. The resulting P values and longitudinal inferences were therefore considered methodologically unreliable.
Fliss-Isakov et al., 2023 [17]	Q1	Yes	The study defined the population receiving CDED and reported criteria that allowed the relevant clinically active adult subgroup to be identified.
Fliss-Isakov et al., 2023	Q2	Unclear	The study was based on real-world clinical data, and the report did not fully establish that disease activity and follow-up outcomes were measured at standardized time points and in an identical, reliable manner for every included participant. Uniform outcome measurement across the cohort could therefore not be confirmed.
Fliss-Isakov et al., 2023	Q3–Q9	Yes	Crohn disease was identified using valid clinical methods, and the study adequately reported participant inclusion, demographic and clinical information, follow-up outcomes and the clinical setting.
Fliss-Isakov et al., 2023	Q10	Yes	The statistical methods used for the reported descriptive and subgroup outcomes were considered broadly appropriate for the observational case-series structure and the available data.
Dileo et al., 2026 [20]	Q1–Q10	Yes	Prospective single-center study with clearly defined eligibility criteria and consecutive recruitment. CD activity and remission were assessed using prespecified clinical and objective criteria. Participant characteristics, intervention, follow-up, attrition, and outcome definitions were clearly reported. Statistical methods were appropriate for the exploratory single-arm design, including explicit ITT handling of non-adherent participants and mixed-effects analyses for longitudinal outcomes.

Q1: Were there clear criteria for inclusion in the case series?

Q2: Was the condition measured in a standard, reliable way for all participants included in the case series?

Q3: Were valid methods used for identification of the condition for all participants included in the case series?

Q4: Did the case series have consecutive inclusion of participants?

Q5: Did the case series have complete inclusion of participants?

Q6: Was there clear reporting of the demographics of the participants?

Q7: Was there clear reporting of clinical information of the participants?

Q8: Were the outcomes or follow-up results clearly reported?

Q9: Was there clear reporting of the presenting sites/clinics' demographic information?

Q10: Was statistical analysis appropriate?

Note: The JBI checklist judgments were interpreted at the item level. No numerical score or overall low-, moderate- or high-quality classification was generated. Concerns regarding population applicability, such as mixed-age recruitment, special treatment history or differences from the review's target PICOS population, were considered separately from the internal methodological appraisal.

Supplementary Figure S2: Traffic-light plot of methodological quality judgments for the included single-arm studies, assessed using the Joanna Briggs Institute Critical Appraisal Checklist for Case Series.

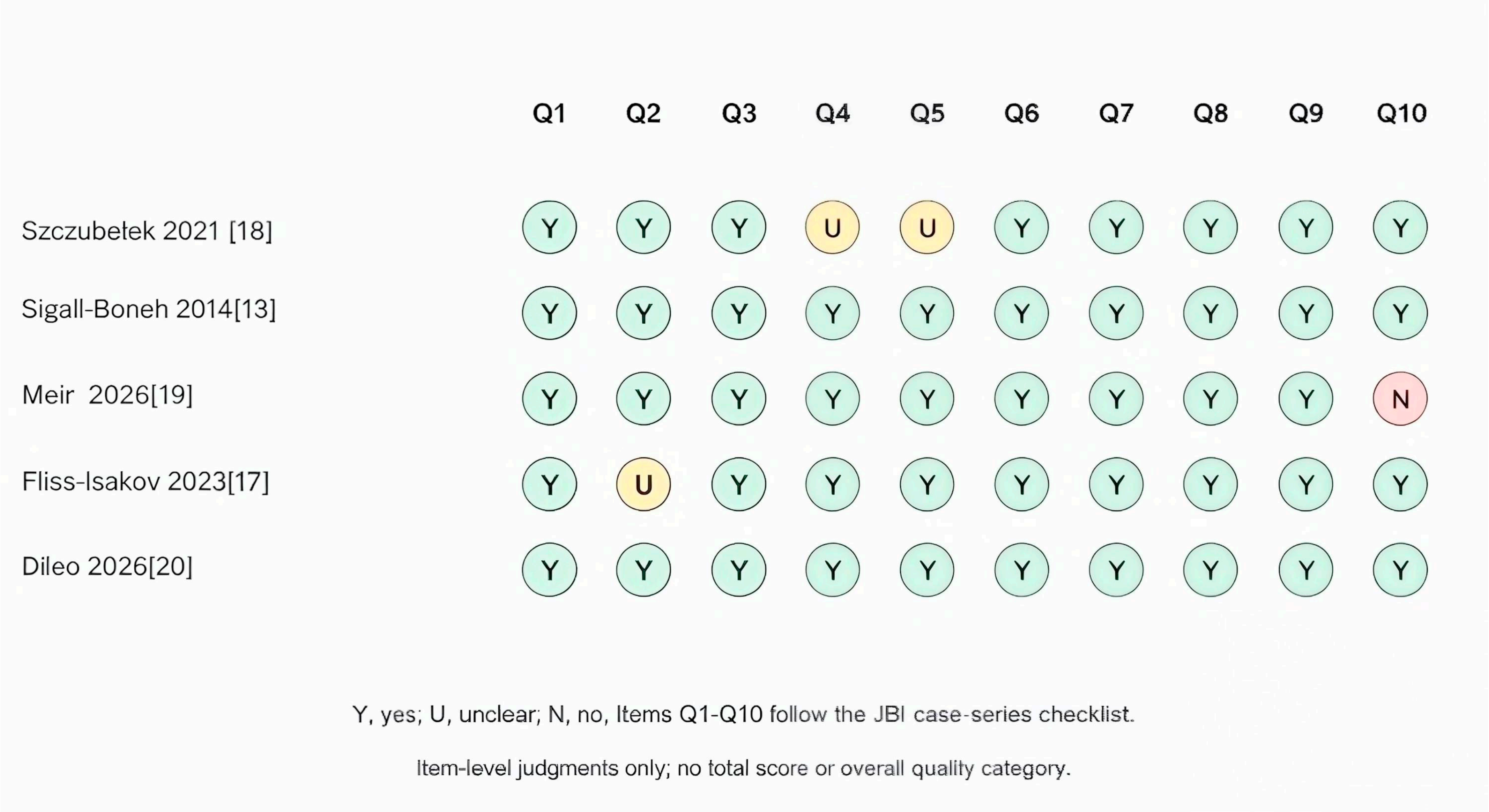

Supplementary Table S6. GRADE certainty of evidence for comparative clinical remission outcomes.

Panel A. CDED plus PEN versus CDED alone (Yanai et al. [15])

Effect estimates

Outcome	Studies/design	CDED+PEN	CDED alone	Relative effect, RR(95% CI)	Absolute effect, RD per 1,000 (95% CI)	Certainty
Clinical remission at Week 6, assessed using HBI <5	1 randomized trial	13/19(68.4%)	12/21(57.1%)	1.20(0.74–1.94)	113 more per 1,000 (185 fewer to 410 more)	Very low ⊕◯◯◯

Certainty-domain judgments

Outcome	Risk of bias	Inconsistency	Indirectness	Imprecision	Other considerations
Clinical remission at Week 6, assessed using HBI <5	Serious[a]	Not assessable[b]	Not serious	Very serious[c]	None

Abbreviations: CI, confidence interval; CDED, Crohn's disease exclusion diet; HBI, Harvey–Bradshaw Index; PEN, partial enteral nutrition; RR, risk ratio; RD, risk difference.

a. Downgraded one level because the open-label design and the use of a symptom-based clinical activity index may have influenced outcome measurement; the overall RoB 2 judgment was high risk.

b. Inconsistency was not assessable because only one study contributed to this outcome; no downgrade was applied.

c. Downgraded two levels because the total sample size was very small and the wide confidence interval included both no important benefit and a potentially substantial benefit.

Publication bias could not be formally assessed because only one trial contributed to each comparison; no additional downgrade was applied.

Panel B. CDED versus the Mediterranean diet (Pasta et al. [16])

Effect estimates

Outcome	Studies/design	CDED	Mediterranean diet	Relative effect, RR(95% CI)	Absolute effect, RD per 1,000 (95% CI)	Certainty

Outcome	Studies/design	CDED	Mediterranean diet	Relative effect, RR(95% CI)	Absolute effect, RD per 1,000 (95% CI)	Certainty
Clinical remission at Week 12, assessed using HBI <5	1 randomized trial	17/24(70.8%)	8/21(38.1%)	1.86(1.02–3.40)	327 more per 1,000 (51 more to 603 more)	Low ⊕⊕◯◯
Clinical remission at Week 24, assessed using HBI <5	1 randomized trial	19/24(79.2%)	9/21(42.9%)	1.85(1.08–3.15)	363 more per 1,000 (96 more to 630 more)	Low ⊕⊕◯◯

Certainty-domain judgments

Outcome	Risk of bias	Inconsistency	Indirectness	Imprecision	Other considerations
Clinical remission at Week 12, assessed using HBI <5	Serious[a]	Not assessable[b]	Not seriousc	Serious[d]	None
Clinical remission at Week 24, assessed using HBI <5	Serious[a]	Not assessable[b]	Not seriousc	Serious[d]	None

Abbreviations: CI, confidence interval; CDED, Crohn's disease exclusion diet; HBI, Harvey–Bradshaw Index; PEN, partial enteral nutrition; RR, risk ratio; RD, risk difference.

a. Downgraded one level because the trial was open-label and clinical remission was assessed using a symptom-based clinical activity index; the overall RoB 2 judgment was high risk.

b. Inconsistency was not assessable because only one study contributed to this outcome; no downgrade was applied.

c. These ratings concern the broader trial populations. Applicability to the initial HBI 5–8 target remains uncertain; no verified subgroup-specific estimate is available.

d. Downgraded one level because the total sample size was small and the confidence interval ranged from a minimal effect to a large benefit.

Publication bias could not be formally assessed because only one trial contributed to each comparison; no additional downgrade was applied.

Supplementary Table S7. Full-text reports excluded after eligibility assessment and reasons for exclusion

Reports and context

Study	Full-text report	Population / intervention context
Sigall Boneh et al., 2017 [28]	Dietary Therapy With the Crohn's Disease Exclusion Diet is a Successful Strategy for Induction of Remission in Children and Adults Failing Biological Therapy. J Crohns Colitis. 2017;11:1205–1212.	Mixed pediatric/adult cohort with loss of response to biologic therapy; CDED+PEN was used as a salvage strategy while biologic treatment was continued.
Levine et al., 2019 [8]	Crohn's Disease Exclusion Diet Plus partial enteral nutrition Induces Sustained Remission in a Randomized Controlled Trial. Gastroenterology. 2019;157:440–450.e8.	Randomized trial comparing CDED+PEN with EEN in children with mild-to-moderate active luminal CD. The trial explicitly enrolled a pediatric population.
Wall et al., 2024 [29]	Preoperative Crohn's Disease Exclusion Diet and Exclusive Enteral Nutrition in Adults with Crohn's Disease: A Feasibility Randomised Controlled Trial. Nutrients. 2024;16:2105.	Adults scheduled for elective CD surgery were randomized to 6 weeks of preoperative EEN, CDED, or usual care; the trial primarily evaluated feasibility and perioperative nutritional/surgical outcomes.
Sigall Boneh et al., 2024 [27]	Cultural Perspectives on the Efficacy and Adoption of the Crohn's Disease Exclusion Diet across Diverse Ethnicities: A Case-Based Overview. Nutrients. 2024;16:3184.	Case-based, multinational overview focused on cultural adaptation and practical implementation of CDED. The authors state that statistical analyses were not feasible and that the work was intended to share experiential observations rather than establish broad efficacy conclusions.
Wijers et al., 2025 [30]	Patient experiences with and adherence to Crohn's disease exclusion diet in Dutch Crohn's disease patients: a cohort study. Ther Adv Gastroenterol. 2025;18:17562848251323553.	Mixed cohort of 69 patients, including 52 pediatric and 17 adult patients; study focus was patient experience, feasibility and adherence to CDED+PEN.
Yin J et al., 2025 [31]	Application of CDED combined with partial enteral nutrition to improve nutritional status and quality of life in patients with Crohn’s disease [translated title; in Chinese]. Xiandai Xiaohua Ji Jieru Zhenliao. 2025;30(2):189–195. doi:10.3969/j.issn.1672-2159.2025.02.013.	86 adults randomized to CDED+PEN or conventional home enteral nutrition (HEN) for 12 months; outcomes were assessed at 3, 6 and 12 months and focused on nutritional status, quality of life, endoscopic scores and inflammatory markers.

Exclusion decisions as supplied

Study	Primary reason for exclusion	PRISMA exclusion category
Sigall Boneh et al., 2017 [28]	CDED+PEN was evaluated as a salvage/adjunctive intervention after biologic treatment failure, rather than the prespecified CDED induction strategy in the target adult mild-to-moderate population.	Ineligible intervention/context
Levine et al., 2019 [8]	Study population did not meet the prespecified adult eligibility criterion.	Ineligible population

Study	Primary reason for exclusion	PRISMA exclusion category
Wall et al., 2024 [29]	CDED was used as preoperative nutritional optimization in a surgical population rather than as the prespecified remission-induction intervention in the target luminal CD population.	Ineligible intervention/context
Sigall Boneh et al., 2024 [27]	No eligible adult induction cohort with an extractable denominator and prespecified clinical remission outcome.	No extractable eligible outcome data
Wijers et al., 2025 [30]	Adult induction remission data meeting the review's prespecified disease-activity definition, remission definition and analysis denominator were not separately extractable for the target quantitative synthesis.	No extractable eligible outcome data
Yin J et al., 2025 [31]	Comparator and intervention framework did not match the prespecified comparative induction evidence structure; conventional HEN was not an eligible comparator for the primary comparative synthesis.	Ineligible comparator

Abbreviations: CD, Crohn’s disease; CDED, Crohn’s disease exclusion diet; EEN, exclusive enteral nutrition; HEN, home enteral nutrition; PEN, partial enteral nutrition.

Note: When more than one eligibility criterion was potentially unmet, a single primary reason for exclusion was assigned according to the prespecified PICOS hierarchy. The categories correspond to those reported in the PRISMA 2020 flow diagram.

Supplementary Table S8. Comparison of the present review with previous systematic reviews of dietary therapy and CDED in Crohn's disease

Review	Literature search / cutoff	Population and scope	Studies included	Synthesis strategy	Main interpretation
Correia et al., 2024 [9]	Publications from 30 June 2014 to 2 August 2022	Children and adults with active CD; specifically CDED/CDED+PEN	9	Systematic review; no quantitative meta-analysis	CDED+PEN showed promising remission and tolerability findings and was considered a potentially viable induction approach, but evidence included mixed age groups and limited studies.
Zhang et al., 2024 [11]	Search conducted 11 November 2023	Pediatric and adult CD; active, mildly active or inactive disease; multiple solid-food dietary interventions, not limited to CDED	14	Structured systematic review; no pooled overall effect; single-arm studies excluded	Solid-food dietary therapy showed promising findings, including CDED in adults, but evidence was limited by small samples and moderate-to-high risk of bias.
Batra et al., 2026 [12]	Search conducted 10 February 2025	Pediatric and adult CDED-based interventions, with a separate adult subgroup of four studies and 134 participants.	17, of which 16 contributed quantitative data	Meta-analysis of pooled remission/response proportions and comparative ORs; subgroup analyses by CDED regimen and comparator	Adult subgroup remission was 64% (95% CI 56%–72%). Overall induction and maintenance syntheses combined broader treatment strategies; well-designed RCTs remained a priority.

Review	Literature search / cutoff	Population and scope	Studies included	Synthesis strategy	Main interpretation
Present review	Search through 2 September 2026	Adult CDED evidence; initial narrow activity target and broader study populations distinguished explicitly	7	Comparator-specific RRs at separate time points; uncontrolled study proportions with exact binomial intervals; study-level missing-outcome scenario; no pooled remission proportion.	Updated adult evidence distinguishes symptomatic remission from objective inflammatory control and links each estimate to the enrolled or assessed population.

Abbreviations: CD, Crohn's disease; CDED, Crohn's disease exclusion diet; EEN, exclusive enteral nutrition; MD, Mediterranean diet; PEN, partial enteral nutrition; RCT, randomized controlled trial.

Note: Previous reviews differed substantially in population scope, eligible dietary interventions, study-design inclusion criteria, and quantitative synthesis strategy. The present review restricts the evidence base to adult CDED studies and preserves differences in comparator, assessment time point, and analysis population rather than pooling clinically distinct estimands. Study counts refer to the number of reports or studies included in each review as reported by the respective authors.

Supplementary Table S9. Individual uncontrolled week-6 remission estimates and denominator sensitivity

Study / scenario	Events / total	Clinical remission (%)	Exact 95% CI (%)	Analysis population	Interpretation
Fliss-Isakov 2023 [17]	30/48	62.5	47.4–76.0	Baseline clinically active subgroup, HBI >=5	Individual descriptive estimate
Szczubełek 2021 [18]	23/30	76.7	57.7–90.1	Available cases, baseline CDAI >150	Individual descriptive estimate
Szczubełek 2021 [18], conservative scenario	23/32	71.9	53.3–86.3	Same study, two missing assessments counted as non-remission	Post hoc study-level denominator sensitivity; not an independent study
Dileo 2026 [20]	22/36	61.1	43.5–76.9	Reported full-cohort ITT, broader activity eligibility	Individual descriptive estimate
Sigall-Boneh 2014 [13], adults	9/13	69.2	38.6–90.9	Adult subgroup, stricter remission threshold HBI <=3	Separate adult contextual estimate

Intervals use the Clopper–Pearson exact binomial method. All uncontrolled estimates are descriptive and are not pooled. The 23/32 row is a post hoc denominator assumption for the same Szczubełek study, not an independent cohort. Clinical remission uses the source-specific HBI or CDAI threshold. Exact intervals quantify sampling uncertainty and do not adjust for selection or confounding.

Supplementary Table S10. Full study characteristics and treatment-context fields

These full fields complement the condensed main Table 1 and retain disease duration, treatment history, and detailed dietary delivery from the source extraction.

Panel A. Full population and disease characteristics

Study	Design	Population / key context	Disease duration	Intervention / comparator
Yanai 2022 [15]	Open-label pilot RCT	Adults aged 18–55 years with active mild-to-moderate, uncomplicated CD; biologic-naïve; maximum permitted disease duration 5 years	CDED+PEN: 3 months (IQR 2–15); CDED alone: 3 months (IQR 1–9)	CDED+PEN (n=19) vs CDED alone (n=21)
Pasta 2025 [16]	Open-label RCT	Adults aged 18–80 years with mild-to-moderate active CD (HBI 5–16)	NR	CDED (n=24) vs Mediterranean diet (n=21)
Fliss-Isakov 2023 [17]	Retrospective real-world cohort	Heterogeneous adult CD population; 72 patients had clinically, biochemically and/or endoscopically active disease; the study-level clinical-remission estimate used the 48 patients with HBI ≥5	Active-disease cohort (n=72): 7.0 ± 8.0 years	CDED with individualized use of PEN and/or concomitant treatment
Szczubełek 2021 [18]	Prospective single-arm study	Adults with active CD (CDAI >150); patients receiving stable maintenance pharmacotherapy, including biologics, could be enrolled	NR	CDED+PEN

Study	Design	Population / key context	Disease duration	Intervention / comparator
Sigall-Boneh 2014 [13]	Prospective case series	Mixed cohort of 34 children and 13 adults; adult subgroup consisted of patients with previous treatment failure and used a stricter remission definition (HBI ≤3)	Adult subgroup: 3.9 ± 4.9 years	CDED+PEN or CDED alone
Meir 2026 [19]	Retrospective real-world cohort	Adults >60 years; 40 patients, of whom 24 were in clinical remission and 16 had active disease at baseline	8.7 ± 11.4 years	CDED with individualized PEN when indicated
Dileo 2026 [20]	Prospective single-center cohort	Adults aged 18–75 years with histologically confirmed active CD; activity defined by HBI >4 plus objective inflammation (CRP >5 mg/L and/or FCP >250 µg/g), or active endoscopic/imaging findings; symptomatic/obstructive strictures, complex fistulizing disease requiring surgery, recent/planned surgery, recent biologic/small-molecule modification, and current systemic corticosteroid use were excluded.	4.0 years (IQR 1.0–14.0)	CDED+PEN (n=36), 52 weeks

Panel B. Full treatment and dietary delivery

Study	Prior biologic exposure	Stable / concomitant background medication	CDED phases	PEN contribution to energy intake

Study	Prior biologic exposure	Stable / concomitant background medication	CDED phases	PEN contribution to energy intake
Yanai 2022 [15]	None; biologic-naïve by eligibility criteria. Current or previous biologic use was excluded.	Concurrent immunomodulators and systemic corticosteroids were excluded. Four patients were receiving stable mesalamine at entry; two continued it during the first 12 weeks.	Phase 1: weeks 0–6; Phase 2: weeks 7–12; Phase 3/maintenance: weeks 13–24	CDED+PEN arm: 1000 kcal/day during weeks 0–6; 600 kcal/day during weeks 7–12; PEN no longer required from week 13, although 600 kcal/day could be continued voluntarily. Percentage of total energy was NR.
Pasta 2025 [16]	Previous biologic exposure was NR as a separate variable; ongoing biologic therapy was permitted and was common at baseline (adalimumab, ustekinumab or vedolizumab).	Biologic or conventional immunosuppressive therapy had to have been initiated ≥24 weeks before enrollment; concomitant corticosteroids and antibiotics were excluded.	Phase 1: weeks 1–6; Phase 2: weeks 7–12; maintenance phase: weeks 13–24	PEN provided 10%–20% of total energy only in patients at nutritional risk according to NRS-2002; 4/24 CDED and 2/21 control participants received PEN.
Fliss-Isakov 2023 [17]	In the broader active-disease cohort (n=72): biologic-naïve 39/72 (54.2%), past biologic therapy 4/72 (5.6%), current biologic therapy 29/72 (40.3%).	Real-world treatment was heterogeneous; CDED could be used alone, as adjunctive or bridge therapy, or together with other therapy. No protocol-wide requirement for a stable pharmacologic background was reported.	Outcomes documented at end of Phase 1 (week 6), Phase 2/end of induction (week 12), and Phase 3 (week 24); some patients with remission/mild disease commenced directly at Phase 2/3.	PEN was prescribed individually according to dietitian assessment rather than a fixed protocol; energy percentage NR. In the clinically active HBI≥5 subgroup, 21/48 received PEN.
Szczubełek 2021 [18]	Some patients were receiving biologic maintenance therapy and had lost response; the exact number with previous biologic exposure was NR.	5-ASA, azathioprine, methotrexate or biologic treatment was permitted only when administered at a fixed dose for >8 weeks; glucocorticosteroids and antibiotics were excluded.	Phase 1: weeks 0–6, restrictive CDED; Phase 2: weeks 7–12, less restrictive diet; after week 12 no structured CDED was prescribed	50% of calculated energy intake from Modulen during weeks 0–6, reduced to 25% during weeks 7–12; smaller amounts/no formula were permitted if patients refused the prescribed amount.

Study	Prior biologic exposure	Stable / concomitant background medication	CDED phases	PEN contribution to energy intake
Sigall-Boneh 2014 [13]	All 13 adults had failed previous medical therapy; the adult-specific number previously exposed to biologics was NR. In the overall cohort, 5 patients had failed or lost response to biologic therapy.	Stable maintenance thiopurine/methotrexate therapy was permitted; stable biologic therapy after failure/loss of response was permitted if dose and schedule remained unchanged. Concurrent remission-induction steroids, 5-ASA, antibiotics or induction-dose biologic therapy were excluded.	Restricted Phase 1: weeks 0–6; step-down Phase 2: weeks 7–12; after week 12 patients were advised to continue self-restriction without a structured diet.	Up to 50% of calories from polymeric formula during weeks 0–6 (maximum 1250 kcal/day), reduced to 25% during weeks 7–12; patients unable/unwilling to take formula could follow CDED without PEN.
Meir 2026 [19]	None: 25/40 (62.5%); current biologic treatment: 12/40 (30.0%); past biologic treatment: 3/40 (7.5%).	Real-world concomitant treatment was permitted; at baseline 7/40 were receiving budesonide and 1/40 prednisone. A protocol-wide medication-stability requirement was NR.	34/40 (85%) started at Phase 1, 3/40 (7.5%) at Phase 2 and 2/40 (5%) at Phase 3; phase unknown for 1 patient. Standard timing was Phase 1 weeks 0–6, Phase 2 weeks 7–12 and Phase 3 from week 13.	PEN was recommended to 12/40 (30%), mainly because of malnutrition/weight loss, low BMI or restrictive phenotype; percentage of total energy NR.
Dileo 2026 [20]	Biologic therapy initiated >3 months before enrollment: 2/36 (5.5%); initiation or modification of biologic/small-molecule therapy within 3 months before enrollment was excluded.	Aminosalicylates 28/36 (77.8%), immunomodulators 2/36 (5.5%), biologic therapy started >3 months before enrollment 2/36 (5.5%); current systemic corticosteroids were excluded.	Phase 1: weeks 0–6; Phase 2: weeks 7–12; Phase 3/long-term maintenance: weeks 13–52.	50% of total energy expenditure during weeks 0–6; 25% during weeks 7–52, using Modulen IBD.

Supplementary Table S11. Report-level cohort provenance and possible patient overlap

Report	Design / center	Recruitment period	Age / clinical population	Overlap assessment and use
Yanai 2022 [15]	Multicenter randomized trial in Israel, including shared investigators with the Tel Aviv reports	12 January 2017–11 May 2020	18–55 years; active, relatively early uncomplicated CD	A different design or recruitment setting does not establish patient-level independence from Fliss-Isakov. Trial-specific effects retained separately.
Fliss-Isakov 2023 [17]	Retrospective cohort, Tel Aviv Medical Center	2018–2021	Adult real-world cohort; clinical-remission denominator restricted to baseline HBI ≥5	Shared center and overlapping period with Meir; possible overlap. No combined participant total or independent-replication claim.
Meir 2026 [19]	Retrospective older-adult cohort, Tel Aviv Medical Center	January 2018–April 2024	Age >60 years; active and remission populations at entry	Possible overlap with Fliss-Isakov. Different age entry criteria from Yanai do not alone prove independence across time; contextual outcomes retained separately.

Assessment uses published report-level information. Individual-level linkage was unavailable and study authors were not contacted. Separate presentation avoids mathematical aggregation across these reports but cannot establish independent recruitment. Sources are main references [15,17,19].

Public source locations: Yanai, https://pubmed.ncbi.nlm.nih.gov/34739863/ ; Fliss-Isakov, https://pmc.ncbi.nlm.nih.gov/articles/PMC10455757/ ; Meir, https://karger.com/ddi/article-pdf/doi/10.1159/000548931/4455406/000548931.pdf .
